# Cell Death Pathway Regulation by Functional Nanomedicines for Robust Antitumor Immunity

**DOI:** 10.1002/advs.202306580

**Published:** 2023-11-20

**Authors:** Yongjuan Li, Yichen Guo, Kaixin Zhang, Rongrong Zhu, Xiaoyuan Chen, Zhenzhong Zhang, Weijing Yang

**Affiliations:** ^1^ School of Pharmaceutical Sciences Henan Key Laboratory of Targeting Therapy and Diagnosis for Critical Diseases Zhengzhou University Zhengzhou Henan 450001 China; ^2^ Medical Research Center The First Affiliated Hospital of Zhengzhou University Zhengzhou University Zhengzhou Henan 450001 China; ^3^ The center of Infection and Immunity Academy of Medical Sciences Zhengzhou University Zhengzhou Henan 450001 China; ^4^ Departments of Diagnostic Radiology, Surgery Chemical and Biomolecular Engineering, and Biomedical Engineering Yong Loo Lin School of Medicine and Faculty of Engineering National University of Singapore Singapore 119074 Singapore; ^5^ Clinical Imaging Research Centre Centre for Translational Medicine Yong Loo Lin School of Medicine National University of Singapore Singapore 117599 Singapore; ^6^ Nanomedicine Translational Research Program NUS Center for Nanomedicine Yong Loo Lin School of Medicine National University of Singapore Singapore 117597 Singapore

**Keywords:** apoptosis and cuproptosis, autophagy and necroptosis, cancer immunotherapy, ferroptosis and pyroptosis, nanomedicines

## Abstract

Cancer immunotherapy has become a mainstream cancer treatment over traditional therapeutic modes. Cancer cells can undergo programmed cell death including ferroptosis, pyroptosis, autophagy, necroptosis, apoptosis and cuproptosis which are find to have intrinsic relationships with host antitumor immune response. However, direct use of cell death inducers or regulators may bring about severe side effects that can also be rapidly excreted and degraded with low therapeutic efficacy. Nanomaterials are able to carry them for long circulation time, high tumor accumulation and controlled release to achieve satisfactory therapeutic effect. Nowadays, a large number of studies have focused on nanomedicines‐based strategies through modulating cell death modalities to potentiate antitumor immunity. Herein, immune cell types and their function are first summarized, and state‐of‐the‐art research progresses in nanomedicines mediated cell death pathways (e.g., ferroptosis, pyroptosis, autophagy, necroptosis, apoptosis and cuproptosis) with immune response provocation are highlighted. Subsequently, the conclusion and outlook of potential research focus are discussed.

## Graphical abstract

This review provides a comprehensive overview of the nanotechnology assisted PCD routes resulting in antitumor immunity including ferroptosis, pyroptosis, autophagy, necroptosis, apoptosis and cuproptosis, systematically introduces their therapeutic mechanisms, nanocarrier types as well as immune cell types, highlights the challenges and perspectives.

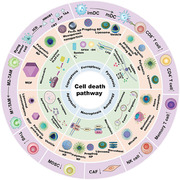

## 1 Introduction

Cellular death of cancer can be categorized as accidental death (ACD) and programmed cell death (PCD) in consideration that whether it is affected by drug or gene.^[^ ^1^ ^]^ ACD is a kind of biological process that is an immediate and unavoidable modality. PCD belongs to signal transduction pathway affected by pharmacologic or genetic intervention. According to the distinct mechanisms, biological features, cell morphologies as well as immunological properties, PCD can be subdivided into ferroptosis, pyroptosis, autophagy, necroptosis, apoptosis and cuproptosis. A variety of factors can initiate PCD, such as intracellular oxidation‐reduction homeostasis destruction, caspase signal activation, gasdermin (GSDM) family evocation and so on.^[^ ^2^ ^]^ A large number of therapies are used in regulating cancer cell death modalities for antitumor response, indicating the emphasis of PCD during cancer treatment.^[^ ^3^ ^]^ However, the limited therapeutic efficacy is observed by directly using PCD inducer or inhibitor due to their fast excretion, low bioavailability and limited tumor accumulation.^[^ ^4^ ^]^ With the development of nanotechnology, nanomedicines have been widely applied into PCD regulation for antitumor treatment to cope with the above issues due to their small size effect, large specific surface area, etc. By virtue of the enhanced permeability and retention effect (EPR), nanomedicines can effectively accumulate in the tumor site to improve therapeutic efficacy and reduce side effects.^[^ ^5^ ^]^ Moreover, because of their physical and chemical properties such as sound, electricity, light, magnetic and thermal responsiveness, nanomedicines can precisely target tumor and synergistically combine with other therapeutic strategies to form functionalized drug delivery systems.^[^ ^6^ ^]^ Compared with traditional anticancer drugs, nanomedicines mainly have the following advantages: 1) Increase the solubility of drugs and improve their stability in vivo, thus reducing drug dosages simultaneously improving therapeutic efficacy. 2) Achieve precision therapy through active/passive targeting. 3) Reduce the blocking effect of physiological barriers to drugs and prolong their blood circulation time in vivo. 4) Facilitate the controlled drug release in vivo by construction of stimulus‐responsive nanosystems. 5) Achieve multi‐functional and integrated diagnosis and therapy.

Host immune system including innate and adaptive immune cells mainly suppresses tumor propagation, metastasis and recurrence.^[^ ^7^ ^]^ However, tumor cells can evade immunosurveillance due to the immunosuppressive microenvironment and low immunogenicity.^[^ ^8^ ^]^ Immunotherapy stimulates the immune system to treat cancer via different strategies including tumor microenvironment modulation, immune checkpoint blockade and vaccine.^[^ ^9^ ^]^ Especially, the discovery of immune checkpoint blockade makes the breakthrough in cancer immunotherapy which can reinvigorate cluster of differentiation 8 positive (CD8^+^) T cells for function restoration.^[^ ^10^ ^]^ CD8^+^ T lymphocytes are generally thought to cause cancer cell lethality by granzyme B (GZMB) or perforin secretion.^[^ ^11^ ^]^ Recently, it has been reported that CD8^+^ T cells could cause tumor ferroptosis via secreted interferon‐γ (IFN‐γ) mediated system x_c_^−^ inhibition.^[^ ^12^ ^]^ PCD including ferroptosis, pyroptosis, autophagy, necroptosis, apoptosis and cuproptosis can elicit host immunity via immunogenic cell death (ICD) when damage‐associated molecular patterns (DAMPs) are released from dying tumor cells for pattern recognition receptors (PRRs) identification in antigen‐presenting cells.^[^ ^13^ ^]^ In addition, as been reported, cuproptosis can upregulate programmed death ligand‐1 (PD‐L1) to increase the sensitivity of anti‐PD‐L1, resulting in potent antitumor immunity.^[^ ^14^ ^]^ The intrinsic relationship between cell death pathway and immunity attracts more and more researchers engaging in PCD mediated cancer treatment.

**Figure 1:**
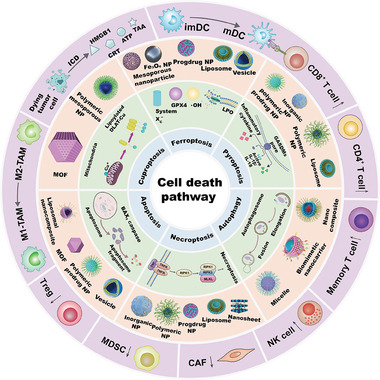
Schematic illustration of nanotechnology‐mediated cell death pathway with antitumor immune response. This review provides a systematic overview of immune cell types (dendritic cell (DC), CD8^+^ T cell, CD4^+^ T cell, memory T cell, natural killer (NK) cell, cancer‐associated fibroblast (CAF), myeloid derived suppressor cell (MDSC), regulatory T cell (Treg), tumor‐associated macrophage (TAM)), and cell death pathways including their types (ferroptosis, pyroptosis, autophagy, necroptosis, apoptosis, cuproptosis), mechanisms, and nanocarriers that to be used. Immunogenic cell death (ICD) process is also presented for dying tumor cells with tumor‐associated antigen (TAA), High Mobility Group Box 1 (HMGB1), adenosine triphosphate (ATP) release and calreticulin (CRT) exposure.

Even though a large amount of work has been reported in tumor treatment focusing on nanomedicines mediated cell death pathways, the systematic review to summarize the recent advances is lacking. Herein, immune cells, cell death pathway types including ferroptosis, pyroptosis, autophagy, necroptosis, apoptosis and cuproptosis mediated by nanotechnology are introduced in detail (**Figure** **1**). Subsequently, conclusions and outlook about cell death pathway mediated antitumor immune response are discussed. We hope that this review can offer some worthy information via analyzing each PCD pathway for precise antitumor immunotherapy and multiple therapeutic approaches combination.

## 2 Immune Cell Types

The innate immunity is a part of the evolution of the immune system.^[^ ^15^ ^]^ The innate immune cells mainly include dendritic cells (DCs), natural killer (NK) cells, *etc*.^[^ ^16^ ^]^ When the innate immune system encounters pathogens, the innate immune cells will react immediately to kill the pathogens and remove them from the host.^[^ ^17^ ^]^ They produce a non‐specific immunity that exists at birth. The ability of the immune system to produce specific cellular responses to pathogens is called adaptive immunity. Innate immune cells respond quickly, while adaptive immune cells have a delayed response that may take several days to fully activate, but can form an immune memory.^[^ ^18^ ^]^ The adaptive immune cells mainly include T and B lymphocytes, which are activated and proliferated by antigen stimulation to produce specific immune response.^[^ ^15^ ^]^ In addition to cancer cells, there also exist immunosuppressive cells such as tumor‐associated macrophages (TAMs), regulatory T cells (Tregs), myeloid‐derived suppressor cells (MDSCs) and cancer‐associated fibroblasts (CAFs) in TME.^[^ ^19^ ^]^ These components transform TME into an immunosuppressive one supporting tumor progression and metastasis, and damage the function of infiltrating APCs and T cells, resulting in the resistance and tolerance of immunotherapy.^[^ ^19e^ ^]^

Different cell subsets emerge different sensitivities to PCD. For example, CD8^+^ T cells can secret IFN‐γ, which down‐regulates the expression of SLC3A2 and SLC7A11 on tumor surface, thereby reducing the intracellular uptake of cystine, and ultimately leading to ferroptosis.^[^ ^12^ ^]^ Knockout of glutathione peroxide 4 (GPX4) in B1 and marginal‐zone (MZ) B cells can trigger their ferroptosis by inducing lipid peroxidation, thus affecting host immune response.^[^ ^20^ ^]^ However, knocking out GPX4 in follicular B cells (FOB) doesn't cause ferroptosis. The possible mechanism is that the content of fatty acid transporter CD36 on the plasma membrane of B1 and MZ B cells is significantly higher than that of FOB cells, resulting in more acid and lipid droplets and thus more prone to lipid peroxidation. In addition, similar to B cells, different subpopulations of macrophages also have different sensitivities to ferroptosis. Compared to anti‐inflammatory M2 macrophage, the higher level of inducible nitric oxide synthase (iNOS) in proinflammatory M1 phenotype elevates the content of nitric oxide free radicals that inhibits lipid peroxidation.^[^ ^21^ ^]^ Therefore, RSL3 as an inhibitor of GPX4 cannot induce M1 macrophage ferroptosis, but does in M2 macrophages. Moreover, immune‐activated cells generally need to inhibit PCD, while immunosuppressive ones need to induce PCD. One advantage of nanomedicines is that it can precisely target cells, so it is important to have a clear understanding of the role and characteristics of each cell.

## 3 Ferroptosis Potentiated Antitumor Immunity

In 2012, Dixon et al. first proposed the concept of ferroptosis, which is an iron‐dependent, non‐apoptotic cell death modality.^[^ ^22^ ^]^ Ferroptosis is mainly caused by the imbalance between generation and degradation of intracellular lipid peroxide (LPO), which presents iron‐mediated oxidative stress elevation and LPO accumulation. A variety of compounds (e.g., erastin, Ras selective lethal 3 compound (RSL3), iron ion, ferroptosis inducing 56 (FIN56)) are able to induce ferroptosis.^[^ ^2^, ^4^, ^23^ ^]^ Although the agonists facilitate ferroptosis via different signal pathways, they mainly directly or indirectly act on glutathione peroxidase resulting in the cellular antioxidant capacity decrement and reactive oxygen species (ROS) accumulation for further oxidative death.^[^ ^24^ ^]^ Studies have shown that tumor ferroptosis can activate the immune response by inducing ICD with DAMPs release, DC maturation, antigen presentation to CD8^+^ T lymphocytes for proliferation, as well as Tregs suppression.^[^ ^25^ ^]^ IFN‐γ secretion by activated T cells has also been reported to induce tumor ferroptosis via system x_c_^−^ inhibition which builds a relationship between immunotherapy and tumor ferroptosis.^[^ ^12^, ^26^ ^]^ Nowadays, more and more researchers pay attention to ferroptosis cascade with antitumor immune response.

However, majority of ferroptosis agonists are small molecule drugs, which generally have poor water solubility, short circulation time and low tumor accumulation in vivo with insufficient efficacy. Nanosystems with unique physicochemical properties can prolong circulation time and increase tumor accumulation through enhanced permeation and retention effect for a high antitumor activity.^[^ ^27^ ^]^ Thus, nanotechnology‐assisted tumor ferroptosis has been widely developed which reveals a huge potential in strengthening antitumor efficacy (**Table** **1**). Tumor ferroptosis mediated by nanomedicines is introduced in the following part according to signal pathways, such as system x_c_^−^ inhibition, GPX4 inhibition and LPO generation.^[^ ^28^ ^]^

**Table 1:** Summarized strategies for ferroptosis mediated cancer immunotherapy via eliciting tumor ICD.

Formulation	Cargos	Mechanism	Tumor model	Refs.
SRF@ Fe^III^TA	SRF, TA, Fe^III^	SRF mediated GPX4 inhibition, Fe^3+^ offering the cycling of Fe^2+^ via Fenton reaction cascade with LPO generation	4T1	[30]
siProminin2@PSN‐FeNP	Prominin2 siRNA	The iron accumulation increment and exosomal iron efflux inhibition	4T1	[31]
CP	CBD	CBD induced LPO generation, GSH consumption and MDA increment	B16F10, 4T1	[32]
BNP@R	RSL‐3	RSL‐3 mediated GPX4 inhibition	B16F10, 4T1	[13c]
Fe^II^PDA@LAP‐PEG‐cRGD	Fe^II^, LAP	Fe^2+^ mediated LPO generation	B16F10	[33]
ZnP@DHA/Pyro‐Fe	cholesterol derivative of DHA and pyropheophorbide‐iron	Reduced Pyro‐Fe for DHA decomposition to produce ROS	CT26, MC38	[34]
MOF@GOx@MnO_2_@ PEG	GOx, MnO_2_	MnO_2_ mediated GSH downregulation, GOx induced H_2_O_2_ generation, Fe‐MOF facilitated Fenton reaction for LPO generation	4T1	[35]
Pa‐M/Ti‐NC	Ti	Pa and Ti induced H_2_O_2_ elevation for Fe ion mediated Fenton reaction enhancement	B16F10	[36]
TFDD	DOX	The Fenton reaction induced by the ferric ions	4T1	[37]
Ce6‐PEG‐HKN_15_	Ce6	Ce6 mediated ROS generation, Fe^3+^ led to the cycling of Fe^2+^ via Fenton reaction for LPO generation	4T1	[38]
CDC@SRF	SRF	CDC induced intracellular GSH depletion, SRF mediated systemic x_c_^−^ inhibition	4T1	[39]
IMSN‐PEG‐Ti	Ti	IMSN‐PEG induced •OH and O_2_ production and GPX4 down‐regulation	CT26	[40]

SRF: sorafenib; TA: Tannic acid; Prominin2: Pentaspan membrane glycoprotein; CP: Cannabinoid nanoparticles with Poly(I:C); CBD: Cannabinoid; PDA: Polydopamine; LAP: b‐lapachone; DHA: Dihydroartemisinin; Pyro‐Fe: Pyropheophorbide‐iron; GOx: Glucose oxidase; Pa: PD‐1 antibody; M: Membrane; Ti: TGF‐β inhibitor; NC: Nanocluster; TFDD: Tannic acid (TA)‐Fe^3+^‐coated doxorubicin (DOX)‐encapsulated DSPE‐PEG micelle; DOX: Doxorubicin; HKN_15_: Ferritin‐homing peptide; CDC: Cinnamaldehyde dimers, SRF: Sorafenib; IMSN: Iron manganese silicate nanoparticles (IMSN); hydroxyl radical: •OH

In addition, ferroptosis that occurs directly in Tregs can also promote tumor immunotherapy.^[^ ^29^ ^]^ Similarly, MDSC with immunosuppressive functions demonstrates resistance to ferroptosis driven by N‐acylsphingosine aminohydrolase 2 (ASAH2) mediated p53‐heme oxygenase‐1 (HMOX1) axis. LPO is rapidly accumulated in the GPX4‐deficient T cell membrane after T cell activation leading to ferroptosis. Moreover, large amounts of LPO were detected in tumor‐derived CD8^+^ T cells, but not in lymph node derived ones, suggesting that ferroptosis may be a metabolic vulnerability point for tumor‐specific CD8^+^ T cells.^[^ ^24b^ ^]^

### 3.1 Nanomedicines Mediated System x_c_^−^ Inhibition

System x_c_^−^ is a cystine/glutamate antiporter widely distributed in the phospholipid bilayer as the transmembrane protein complex. It is an important part of the cellular antioxidant system and is a heterodimer composed of two subunits SLC7A11 and SLC3A2. System x_c_^−^ mainly transports extracellular cystine into cells to produce cysteine, which is one of the synthetic substrates of antioxidant glutathione (GSH). The metabolic consequence of system x_c_^−^ inhibition is a rapid depletion of intracellular cysteine, which leads to the GSH synthesis obstruction. GSH plays a key role in protecting the cells from oxidative damage by catalytic reduction of harmful hydroperoxides, the lack of which often results in GPX4 inhibition with LPO accumulation, ultimately leading to ferroptosis. Nowadays, system x_c_^−^ has been a targeting point in nanomedicine‐assisted ferroptosis cancer immunotherapy.^[^ ^13^, ^41^ ^]^

Stimulus‐responsive (e.g., pH, GSH) nanomedicines are generally prepared to controlled deliver system x_c_^−^ inhibitors into the focal area for high ferroptosis efficacy. For example, Xie et al. constructed a nanoplatform MPDA@Fe_3_O_4_‐Era with erastin (Era) and Fe_3_O_4_ nanoparticle loading onto the surface of mesoporous polydopamine (MPDA) to amplify intracellular ROS and LPO for synergistic ferroptosis therapy.^[^ ^42^ ^]^ Under the acidic condition in endo/lysosome and local near‐infrared stimulation, Fe_3_O_4_ was released from the nanoplatform and reacted with the intracellular hydrogen peroxide (H_2_O_2_) via Fenton reaction with hydroxyl radical (•OH) generation for further LPO products. Simultaneously, erastin inhibited the system x_c_^−^ pathway with GSH depletion. MPDA@Fe_3_O_4_‐Era plus laser irradiation indicates a highly effective tumor suppression, which raises •OH level, inactivates GPX4, improves small molecule Era bioavailability for potent system x_c_^−^ inhibition and promotes the development of precise synergistic tumor therapy. In another example, Xin et al. constructed a nano prodrug system targeting system x_c_^−^ to amplify the ferroptosis efficacy in cancer.^[^ ^43^ ^]^ They prepared a GSH‐responsive ferroptosis nanoprecursor SSZ‐Fe^2+^@DSSD, which was composed of a disulfide bridged levodopa (DSSD), system x_c_^−^ inhibitor sulfasalazine (SSZ) and Fe^2+^. SSZ‐Fe^2+^@DSSD revealed a good stability under the normal physiological environment, while under a low endo/lysosome pH value and a high intracellular GSH level, its nanostructure was destroyed with SSZ and Fe^2+^ release for system x_c_^−^ inhibition, GPX4 down‐regulation and LPO generation. In vitro results showed that the nano precursor drug significantly destroyed the cellular redox homeostasis. Based on the synergistic effect of SSZ and Fe^2+^, the nano prodrug displayed a significant antitumor effect on xenografted tumor bearing mice.

**Figure 2:**
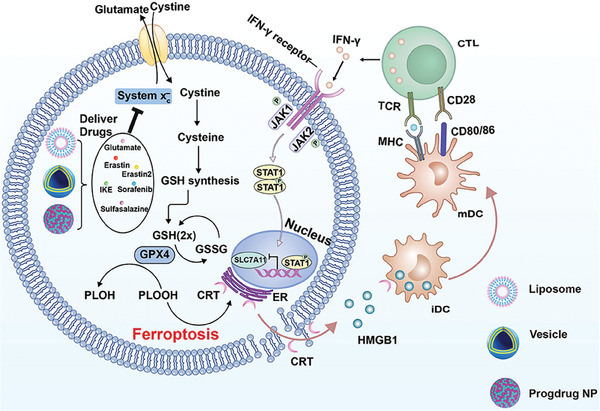
Schematic illustration of nanomedicines mediated system x_c_^−^ inhibition for ferroptosis cascaded cancer immunotherapy. Nanocarriers such as liposome, vesicle and prodrug deliver system x_c_^−^ inhibitors to prevent cystine transportation into cells which impedes GSH synthesis and down‐regulates GPX4 with LPO accumulation for ferroptosis. This process induces tumor ICD with DAMPs release for DC maturation and T cell activation, and the formed CTL secrets IFN‐γ that also inhibits system x_c_^−^ for ferroptosis.

In addition to the direct tumor lethality, ferroptosis can also elicit host immune responses for robust antitumor activity. Sun et al. developed cinnamaldehyde dimers (CDC) to form dimersomes which were abundant with double bonds for intracellular GSH depletion via Michael addition reaction (**Figure** **2**).^[^ ^39^ ^]^ After combination with sorafenib (SRF), a system x_c_^−^ inhibitor, the dimersomes self‐assembly into CDC@SRF led to an enhanced ferroptosis and a robust antitumor immunity with ER stress for calreticulin (CRT) exposure, DC maturation and CD8^+^ T cell priming. The feasible synthesis procedure as well as the similar preparation method with liposome may contribute to the clinical translation of this nanoplatform. Dai et al. prepared a nanounit HGF for immunosuppression reversion when hydrophobic 5 β‐cholanic acid (CA) was conjugated in hyaluronic acid (HA) with exosome inhibitor GW4869 encapsulation and polyphenol conjugation for Fe^3+^ coordination.^[^ ^44^ ^]^ The authors claimed that GW4869 release from HGF decreased tumor‐derived exosome secretion as well as impaired exosomal PD‐L1. This process reinvigorated T cells with reactive IFN‐γ secretion for SLC7A11 and SLC3A2 expression inhibition (Figure 2). In addition, the intracellular Fe^3+^ release from HGF also strengthened tumor ferroptosis. In vivo studies showed that HGF suppressed tumor growth with a long immune memory response. This report subtly exploits the loop of ferroptosis and antitumor immunity to augment therapeutic efficacy.

### 3.2 Nanomedicines Induced GPX4 Inhibition

There are many members in GPXs family including GPX1‐GPX8, among which GPX4 is a key regulator of ferroptosis. It is an inhibitory protein during the lipid peroxidation process, capable of degrading small molecules or complicated peroxides.^[^ ^45^ ^]^ There are several ways to induce tumor ferroptosis via focusing on GPX4, such as directly targeting GPX4, GPX4 enzymatic function inhibition, GPX4 deletion, etc.^[^ ^31^, ^46^ ^]^ GPX inhibitors mainly include RSL3, FIN56, diphenyleneiodonium 7 (DPI7), DPI10 and endoperoxide‐containing 1, 2‐dioxolane (FINO_2_). For example, Stockwell et al. found that FINO_2_ not only indirectly inhibited the enzymatic function of GPX4, but also directly oxidized Fe^2+^, ultimately leading to extensive LPO generation, indicating its multiple ferroptosis mechanisms.^[^ ^47^ ^]^ Nanotechnology as the common drug delivery strategy plays a key role in tumor ferroptosis targeted GPX4.^[^ ^30^, ^48^ ^]^

**Figure 3:**
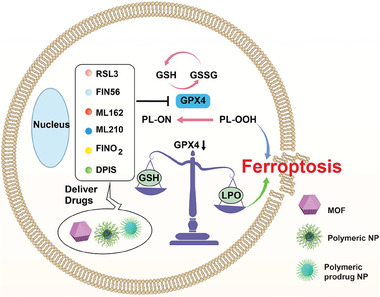
Schematic illustration of nanomedicines mediated GPX inhibition for ferroptosis via delivering GPX inhibitors. Nanomaterials such as MOF, polymeric and prodrug nanoparticles deliver GPX4 inhibitors resulting in LPO accumulation for ferroptosis. In addition, intracellular GSH depletion also reduces GPX4 expression with LPO accumulation.

Sun et al. designed a LPO nanoamplifier using a simple drug delivery‐drug (DDD) engineering strategy, when the stable nanoparticles were co‐assembled from FIN56 and arachidonic acid (AA) (**Figure** **3**).^[^ ^49^ ^]^ Amphiphilic distearyl phosphatidyl ethanolamine‐SS‐polyethylene glycol (DSPE‐SS‐PEG) polymers were modified onto the nanoamplifier surface for long circulation time and a tumor‐specific drug release. AA was found to be converted into PL‐AA in tumor cells through a two‐step reaction catalyzed by long‐chain fatty acid‐coA ligase 4 (ACSL4) and lysophosphatidyl cholinyl transferase 3 (LPCAT3). Subsequently, the intracellular PLs‐AA was oxidized to cytotoxic PL‐AA‐OOH by lipoxygenase (LOXs) which was synergized with FIN56‐mediated GPX4 loss for effective tumor ferroptosis. This study is a conceptual step forward for ferroptosis driven nanotherapies and provides a new co‐delivery paradigm for combined therapy. Liu et al. provided an iron‐supplied regenerative therapy, using Fe^3+^ and natural tannic acid (TA) to spontaneously form a network corona on SRF nanonucleus, named as nano‐SRF@Fe_III_TA (SFT).^[^ ^30^ ^]^ Under acidic lysosomal conditions, SRT dissociated with SRF release and GPX4 enzyme activity inhibition. This research provides an idea in exploiting nanotherapy to induce ferroptosis after the versatile material design.

Nanomedicine based ferroptosis using GPX4 as a target can also elicit antitumor immune response.^[^ ^23^, ^50^ ^]^ Yu et al. designed an intracellular‐acidity‐activatable nanoplatform by integrating ionizable block conjugation with photosensitizer pheophorbide a (PPa) and acid‐liable phenylboronate ester (PBE) to encapsulate GPX4 inhibitor RSL‐3 (Figure 3).^[^ ^13c^ ^]^ After protonation of the ionizable core, the nanoplatform indicated an acid‐activatable photodynamic therapy (PDT) with T lymphocyte recruitment and infiltration accompanied by IFN‐γ secretion, which sensitized RSL‐3‐inducible ferroptosis. When combined with anti‐PD‐L1, the nanoplatform notably inhibited melanoma growth and lung metastasis, indicating the huge potential of ferroptosis in potentiating cancer immunotherapy. In another example, Cai et al. reported a nanoreactor Cu_2_‐_x_Se/ZIF‐8@Era‐PEG‐FA that could enable the conversion of Cu^+^ and Cu^2+^ for oxygen (O_2_) self‐generation and intracellular GSH consumption with GPX4 down‐regulation.^[^ ^48^ ^]^ The loaded Era was able to up‐regulate NADPH oxidase 4 (NOX4) protein expression and increase the intracellular H_2_O_2_ concentration, laying the foundation of O_2_ generation and ferroptosis. Furthermore, the O_2_ generation relieved hypoxia with the miR301 expression decrement in secreted exosomes with TAMs phenotype polarization. The O_2_ self‐supplying nanoreactor subtly combines ferroptosis with TME modulation demonstrating a potential approach for clinical translation due to the feasible synthesis. However, the typical pathway of Era as a system x_c_^−^ inhibitor isn't mentioned in this report which should be further explored about the multiple ferroptosis mechanisms in the future.

### 3.3 Iron‐Based Nanomedicines for Lipid Peroxides Generation

Ferroptosis is an iron dependent cell death pathway when the intracellular iron ion can generate •OH for LPO accumulation in cell membranes.^[^ ^33^, ^51^ ^]^ Iron based nanoformulations including Fe_3_O_4_ nanoparticles and iron ion coordination nanocomplexes are generally used in tumor ferroptosis cascaded cancer immunotherapy.^[^ ^40^, ^50^, ^52^ ^]^

**Figure 4:**
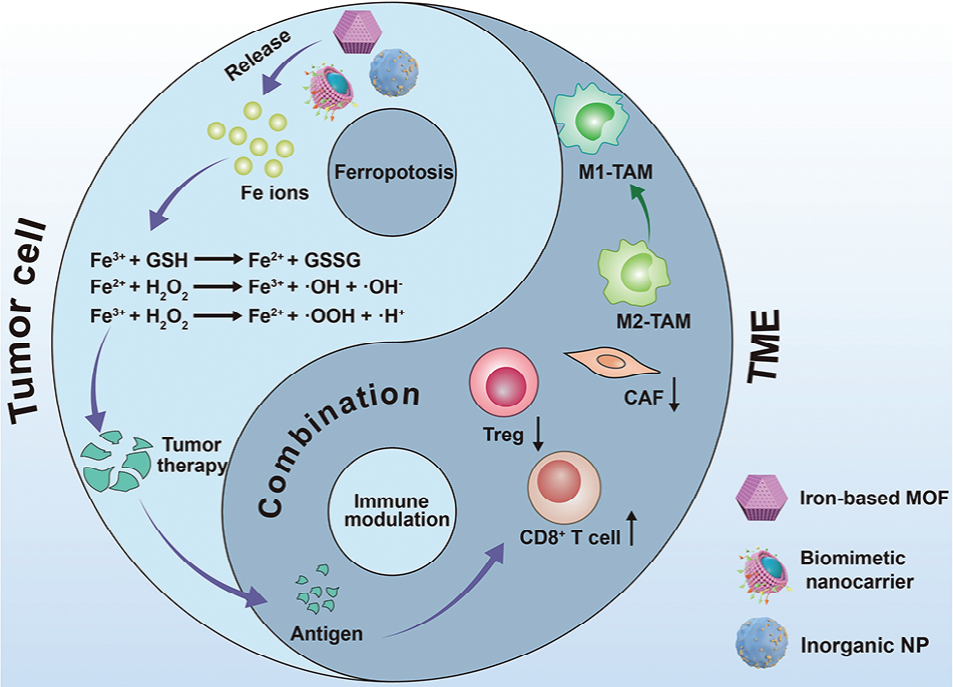
Schematic illustration of iron‐based nanomedicines for ferroptosis via Fenton reaction which combines with other strategies resulting in TME modulation.

Han et al. developed a metal‐organic framework nanoplatform based on iron which was modified with manganese dioxide (MnO_2_), glucose oxidase (GOx), and PEG on surface for redox and iron metabolism homeostasis disruption (**Figure** **4**).^[^ ^35^ ^]^ Fe^2+^ mediated ferroptosis via Fenton reaction could be reinforced by GSH consumption and H_2_O_2_ generation in the presence of MnO_2_ and GOx. The released tumor‐associated antigens via ferroptosis revealed a powerful antitumor immune response after combination with aptamer‐PD‐L1. Mao et al. developed an Ir complex (named as Ir1) with a ferrocene‐conjugated diphosphine ligand for lysosomes localization (Figure 4).^[^ ^53^ ^]^ Due to the low pH value in lysosomes, Ir1 catalyzed Fenton‐like reaction with •OH generation and GPX4 down‐regulation. It also induced tumor ICD via DAMPs release with T cell activity regulation and immune microenvironment modulation. In all, iron‐based nanomedicines via coordination structure reveal a huge potential in ferroptosis‐based cancer immunotherapy, therapeutic efficacy of which can be improved after combination with immune checkpoint blockade. However, the in vivo stability and biocompatibility of coordinated complexes may lead to their safety issues that should not be ignored.

In addition to coordination, Sun et al. reported ultrasmall single‐crystal Fe nanoparticles (bcc‐USINPs) which were highly cytotoxic in the acidic endo/lysosome due to Fe_3_O_4_ shell etching and Fe(0) core exposure.^[^ ^54^ ^]^ They found that bcc‐USINPs elicited potent LPO accumulation with tumor ferroptosis and ICD at a low concentration. In vivo results revealed that iRGD‐bcc‐USINP notably inhibited tumor growth, promoted DC maturation, provoked T lymphocytes and elicited a robust immune response after combination with anti‐PD‐L1. Thus, both Fe^2+^ and Fe(0) can induce ferroptosis‐based cancer immunotherapy, providing more choices in iron‐based nanosystem construction. Iron‐based biomimetic nanosystems offer good biosafety and homing effect that display superior advantages than traditional nanoplatform.^[^ ^55^ ^]^ Xie et al. constructed a biomimetic magnetosome with Fe_3_O_4_ nanoparticle as the core and leukocyte membrane as the cloak, where transforming growth factor‐β (TGF‐β) inhibitor (Ti) was encapsulated inside membranes for TME modulation and PD‐1 antibody (Pa) was anchored on membrane surface for immune checkpoint blockade (Pa‐M/Ti‐NC) (Figure 4).^[^ ^36^ ^]^ Inside the tumor, released Pa and Ti increased its immunogenicity and facilitated M2 phenotype macrophages polarization into M1 ones, which enhanced robust tumor ferroptosis via released Fe^2+^ from nanocarriers mediated Fenton reaction. The cooperation between immunomodulation and ferroptosis generated robust antitumor efficacy with inhibition of both tumor volume growth and lung metastasis. However, how to elevate the intracellular H_2_O_2_ level isn't mentioned in the above report while it is essential to reinforce Fenton reaction mediated ferroptosis.

### 3.4 Nanomedicines Mediated Other Ferroptosis Pathway

Although the traditional activities of p53 (e.g., cell cycle arrest, senescence, apoptosis) are widely believed as major checkpoints in the stress response, growing evidence suggests the importance of its other antitumor mechanisms.^[^ ^56^ ^]^ In these unconventional activities, its ability to induce ferroptosis has attracted tremendous interest.^[^ ^57^ ^]^ Gu et al. found that p53 was able to impede cystine uptake via inhibiting the expression of SLC7A11 leading to tumor ferroptosis.^[^ ^58^ ^]^ p53^3KR^ is an acetyl‐deficient mutant that cannot induce cell cycle arrest, senescence and apoptosis, but completely retains the ability to regulate SLC7A11 expression and induces ferroptosis under ROS stress. By using p53^3KR/3KR^MDM2^−/−^ mutant mice (murine double minute gene named as MDM2), the authors further demonstrated that this aspect of p53 function was important for embryonic development and MDM2 deletion‐related mortality. Thus, ROS‐induced ferroptosis is significantly increased in p53‐activated cells.

Lipoxygenase mostly mediates the formation of iron‐dependent LPO, because it can catalyze the oxidation of polyunsaturated fatty acids (PUFAs) and be inactivated by lipophilic iron chelating agents. The mammalian lipoxygenases family consists of six isoforms (arachidonate lipoxygenase 3 (ALOXE3), arachidonic acid 5‐lipoxygenase (ALOX5), ALOX12, ALOX12B, ALOX15 and ALOX15B) with different substrate specificities. Chu et al. found that ferroptosis was suppressed in ALOX12 knockout U2OS cells and that loss of one ALOX12 allele was sufficient to abolish p53‐mediated ferroptosis and accelerate tumorigenesis, indicating that the ferroptosis pathway mediated by ALOX12 was critical for p53‐dependent tumor suppression.^[^ ^59^ ^]^ Voltage dependent anion channels (VDACs) are transmembrane channels that transport ions and metabolites, and play an important role in the process of iron degradation. Stockwell et al. found that erastin was able to act on VDACs causing mitochondrial dysfunction, which facilitated the release of oxidizing substances for oxidative cell death.^[^ ^60^ ^]^

In addition to the main mechanisms mentioned above, ferroptosis is also regulated by other pathways. For example, heme oxygenase‐1 (HO‐1) is an important source of intracellular iron. Kwon et al. confirmed that HO‐1 was able to induce LPO for tumor ferroptosis.^[^ ^61^ ^]^ Although DOX is generally used in chemotherapy, it can also induce ferroptosis via heme synthesis obstruction leading to iron overload cascade with ferroptosis in mitochondria.^[^ ^62^ ^]^ Doll et al. showed that ferroptosis suppressor protein 1 (FSP1) catalyzed the regeneration of coenzyme Q10 (CoQ_10_) through NAD(P)H, and then combined with GPX4 and GSH to inhibit phospholipid peroxidation.^[^ ^63^ ^]^ Thus, it can increase ferroptosis sensitivity when losing FSP1 ubiquitin by CoQ10 decrement and LPO generation.

## 4 Pyroptosis Integrated Cancer Immunity

Pyroptosis is an inflammatory form of PCD, which is characterized by the formation of pores in the plasma membrane, cell swelling with giant bubbles generation, chromatin fragmentation, and cytoplasmic proinflammatory cytokines extravasation.^[^ ^2^, ^64^ ^]^ It can reshape the immunosuppressive TME, turn the “cold” tumor into an immunogenic “hot” one, and enhance immunotherapeutic efficacy. Pyroptosis is induced by GSDM superfamily, including GSDMA, GSDMB, GSDMC, GSDMD and GSDME.^[^ ^65^ ^]^ The gasdermin‐N‐terminal fragments are intrinsically cytotoxic, which are usually covered by the inhibitory gasdermin‐C‐terminal fragments. Protein between C‐ and N‐terminal segments is hydrolyzed by caspase (e.g., caspase‐1/3/4/5/8/11) resulting in gasdermin cleavage with gasdermin‐N terminus release for plasma membrane transfer. Gasdermin‐N can be transferred to the plasma membrane and destroy the structure of the membrane to form pores with cell permeability destruction. As a result, the plasma membrane is inflated leading to inflammatory cytokines (e.g., IL‐1β, IL‐18) secretion and high mobility group box 1 (HMGB1) release for DC maturation and immune response activation. In human derived cells, the gasdermin family consists of six members: GSDM‐A, ‐B, ‐C, ‐D, ‐E (also known as deafness, autosomal dominant 5 (DFNA5)) and autosomal recessive deafness‐59 (DFNB59). Murine derived cells lack GSDMB but express three GSDMA (GSDMA1‐3) and four GSDMC (GSDMC1‐4).

Pyroptosis is very different from apoptosis. First, DNA damage is different. In contrast to apoptosis, cells undergoing pyroptosis have condensed chromatin and fragmented DNA, but their nuclei remain intact. In addition, pyroptotic cell mediated DNA damage manifests low intensity and the terminal deoxynucleotidyl transferase dUTP nick‐end labeling (TUNEL) staining is positive. Second, swelling and osmotic lysis of pyroptotic cells are caused by inflammation‐mediated pores, the formation of which is dependent on caspase‐1/3/4/5/8/11 activation. We will summarize partially representative examples in **Table** **2** for nanomedicine mediated cancer pyroptosis‐immunotherapy. Gasdermin family mediated pyroptosis‐cancer immunotherapy assisted by nanotechnology will be separately introduced in this section.

**Table 2:** Summarized strategies in pyroptosis inducement for enhanced cancer immunotherapy with DAMPs release.

Formulation	Cargo	Mechanism	Tumor model	Refs.
CA‐Re	Rhenium	CA‐Re PDT exhausted GPX4, resulting in GSDMD cleavage	MDA‐MB‐231, 4T1	[66]
(M+H)@ZIF/HA	Mitoxantrone, hydralazine	Methylglyoxal decrement transformed apoptosis into pyroptosiswith GSDME‐N expression	4T1	[67]
Nano‐CD	cisplatin	A specific sgRNA unlocked GSDME expression with caspase‐3 activation	B16F10	[13d]
PDNP	DOX	DOX provoked GSDME activation	CT26, 4T1	[3c]
TBD‐3C	TBD‐3C	PDT mediated GSDMD cleavage	KPC, Panc02	[68]
CaNMs	Ca^2+^, curcumin	Ca^2+^ overload in mitochondrial for ROS increment and cytochrome C release with caspase‐3 activation to cleave GSDME	4T1	[69]
ZrNPs	K^+^, [ZrF_7_]^3−^	K^+^ and [ZrF_7_]^3−^ ions release resulting in osmolarity elevation and homeostasis imbalance for caspase‐1 activation with GSDMD cleavage	4T1	[70]
NBS‐1MT	NBS, 1‐MT	Caspase‐1 activated by inflammasome with GSDMD cleavage	4T1	[71]
AIE D1	None	AIE D1 produced ROS for caspase‐1 activation with GSDMD cleavage	4T1	[72]
EV^Tx^	mRNA, Ce6	Ce6 mediated puromycin inactivation with GSDMD‐N activation	4T1	[73]
CCNP	Camptothecin, Ce6	ROS production in mitochondria induced caspase‐3 activation with GSDME cleavage	4T1	[74]
COF‐909‐Cu	Cu^2+^	Intracellular H_2_O_2_ level elevation with CDT for caspase‐3 activation with GSDME cleavage	4T1	[51]
DTAP	PF, Flav	DTAP reaction with Casp1 and GGT to turn on its NIRF signal	4T1	[75]
MRC NPs	RGX‐104, Ce6	PDT strengthened oxidative stress and organelles destruction with GSDME cleavage	4T1	[76]
AOZN	Orz, AMPCP	Orz upregulated GSDMD expression, AMPCP induced active caspase‐1 with GSDMD cleavage	B16F10, CT26	[77]
Lmo@RBC	None	ROS mediated by NADPH oxidase for caspase‐8 activation with GSDMC expression	CT26	[78]
OPDEA‐PDCA	None	OPDEA‐PDCA induced mtROS production and oxidative stress with GSDMD cleavage	MNNG‐HOS, K7M2	[79]

CAIX: Carbonic anhydrase IX; Re: Rhenium; Nano‐CD: Nano‐CRISPR scaffold; PDNP: pH‐activated supramolecular nanoprodrug; TBD‐3C: A membrane‐targeted photosensitizer; CaNMs: Ca^2+^ nanomodulators; ZrNPs: K_3_ZrF_7_:Yb/Er upconversion nanoparticles; 1‐MT: 1‐methyltryptophan (1‐MT); AIE: Aggregation‐induced emission; D1: Dimer‐1; EV^Tx^: Extracellular vesicles‐based GSDMD‐N mRNA delivery system; CCNP; Camptothecin prodrug polymer and Ce6 composed in nanoparticles; COF: Covalent organic frameworks; Orz: Oryzanol; AMPCP: α, β‐methylene adenosine 5′ diphosphate; DATP: Dual‐locked and tandem activatable probe; PF: PF‐04691502; Flav: flavopiridol; Camptothecin: CPT; Ce6: chlorine e6; MRC NPs: MRC nanoparticles; AOZN: Nanoparticle with AMPCP and Orz encapsulation; LMO: Listeria monocytogenes; RBC: Red blood cell.

### 4.1 Nanomedicines Induced GADMA Activation

**Figure 5:**
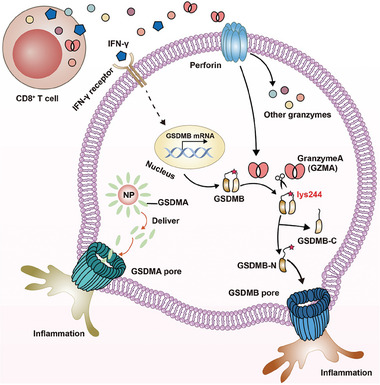
Schematic illustration of GSDMA and GSDMB pathways for inflammatory response. Prodrug nanoplatform induces GSDMA mediated pyroptosis via intracellular GSDMA release for inflammatory response. CD8^+^ T cell secrets IFN‐γ to elevate intracellular GSDMB expression, and simultaneously releases granzyme A to cleavage GSDMB with active GSDMB‐N formation for inflammation response.

Gasdermins are selectively expressed in specific mucosal sites, among which GSDMA is mostly distributed in skin and upper gastrointestinal epithelial cells. Liu et al. found that the cysteine protease streptococcal pyrogenic exotoxin B (SpeB) cleaved GSDMA after Gln246 and formed an N‐terminal (NT) fragment (GSDMA‐NT) to trigger 293T pyroptosis.^[^ ^80^ ^]^ There were two cysteine residue mutations in the C‐terminal domain of GSDMA3, which didn't affect the pyroptosis induced by the complex of N and C domains of GSDMA3. Shao et al. conjugated GSDMA3 (N + C) to nanoparticles using triethylsilyl (TES) ether linker to form NP‐GSDMA3 (**Figure** **5**).^[^ ^81^ ^]^ When NP‐GSDMA3 incubated with Phe‐BF3, GSDMA3 (N + C) was released from NP‐GSDMA3 with holes formation in the liposome membrane. The tumor volume growth was notably inhibited when mice suffered from NP‐GSDMA3 and Phe‐BF3 treatment total three times. In immunodeficient mice or mice with T cell exhaustion, there was negligible change in tumor volume, indicating that pyroptosis mediated tumor regression required CD8^+^ CD4^+^ T cell assistance and was closely related to the host immune system.

### 4.2 GSDMB

Gasdermin B‐mediated pyroptosis depends on secreted Granzyme A (GZMA) from NK and T cells to cleave GSDMB domain (Figure 5).^[^ ^82^ ^]^ Shao et al. found that up‐regulation of IFN‐γ could induce GSDMB expression for apoptosis (Figure 5). When GZMA enters apoptotic cells, it cleaved GSDMB with apoptosis transformation into pyroptosis. In addition, mice inoculated with CT26 containing high GSDMB expression displayed a complete tumor regression after anti PD‐1 treatment, indicating the important role of GSDMB in regulating tumor cell death. GSDMB is highly expressed in epithelial cells of the digestive tract as well as in derived tumors. Among the regulatory elements of GSDMB, there are two different promoters. One is an Alu‐derived promoter, which only directs expression in normal gastric tissues. The other is a long‐terminal‐repeat (LTR)‐derived promoter, directing GSDMB expression in various cancer types and normal tissues. Intracellular GSDMB levels are positively correlated with the survival rate of patients.

### 4.3 Nanomedicines Mediated GSDMC Activation

GSDMC mediated pyroptosis mainly depends on metabolite α‐ketoglutaric acid (α‐KG) to recruit caspase‐8 for GSDMC cleavage. In one example, cells with DM‐Αkg (a cell‐permeable derivative of α‐KG) treatment increased intracellular ROS levels, which resulted in the oxidation of plasma membrane‐localized death receptor 6 (DR6).^[^ ^83^ ^]^ After DR6 oxidation, it entered the cytoplasm to form DR6 receptosome, and DM‐Αkg enhanced DR6 receptors to recruit pro‐caspase‐8 and GSDMC. The DR6 receptor provided a platform for active caspase‐8 to cleave GSDMC, resulting in pyroptosis. Furthermore, α‐KG‐induced pyroptosis inhibited tumor growth and metastasis, efficacy of which depends on the acidic environment. In an acidic environment, α‐KG is reduced and converted to L‐2‐hydroxyglutarate (L‐2HG) by the metabolic enzyme malate dehydrogenase 1 (MDH1), further increasing ROS levels. Considering that pyroptosis can enhance the antitumor immunity, α‐KG‐based therapy may be an effective therapeutic strategy for tumors that are heavily dependent on glycolysis.

In another example, to reduce the side effects (e.g., systematic inflammation) of bacterium Listeria monocytogenes (Lmo), Shen et al. constructed a biomimetic system when red blood cell (RBC) membranes were used to encapsulate Lmo to form Lmo@RBC for GSDMC‐mediated pyroptosis.^[^ ^78^ ^]^ The enhanced tumor accumulation, remodulated TME as well as lasting antitumor immune response were observed for mice after Lmo@RBC treatment. Thus, utilizing living bacterial to build a biomimetic system can serve as a proof‐of‐concept of bacteria vaccine via initiating tumor pyroptosis to potentiate tumor immunotherapy efficacy.

### 4.4 Nanomedicines Induced GSDMD Activation

GSDMD can be cleaved by caspase‐1 or caspase‐11/4/5.^[^ ^84^ ^]^ After cleavage, GSDMD releases a N‐terminal p30 fragment (GSDMD‐N), which binds to membrane phospholipids oligomerizing into a large pore of ≈18 nm at the plasma membrane. The pores serve as channels for IL‐1β and IL‐18 release, which ultimately lead to cell death.^[^ ^85^ ^]^ Ning et al. found that the loss of mixed‐lineage leukemia 4 (MLL4) weakened the strength of classical and super enhancers, thereby weakening the expression of Argonaute 2 (AGO2) and DNA methyltransferase 1 (DNMT1) in the RNA‐induced silencing complex (RISC).^[^ ^86^ ^]^ This process promoted double‐stranded DNA stress, induced GSDMD expression and N‐terminal hydrolysis, and initiated tumor cell pyroptosis. It was found that the transcription level of GSDMD was positively correlated with the mRNA levels of CD3, CD8A, GZMA, GZMB and PRF1. These results indicated that the high expression of GSDMD promoted CD8^+^ T cell infiltration accompanied by high cytotoxic activity. The research also demonstrated that GSDMD had an immunostimulatory effect.

**Figure 6:**
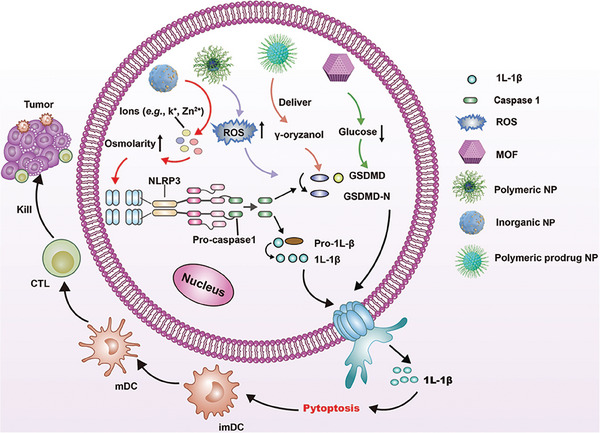
Schematic illustration of nanomedicines mediated GSDMD activated pyroptosis for host antitumor immunity provocation. Nanomedicines, which deliver ions with osmolarity elevation, induce ROS generation, or reduce intracellular glucose concentration can improve GSDMD levels accompanied by GSDMD‐N formation after caspase‐1 cleavage. This process induces pro‐inflammatory factors release for pyroptosis which facilitates DC maturation and T cell activation to form CTL resulting in tumor cell death.

Recently, a large number of studies have been reported that nanotechnology‐based pyroptosis can induce antitumor immune response.^[^ ^3^, ^79^, ^87^ ^]^ For example, Lin et al. developed biodegradable upconversion nanoparticles K_3_ZrF_7_:Yb/Er (denoted as ZrNPs) which could be dissolved inside cancer cells and release a large amount of ions like K^+^ and [ZrF7]^3−^ for a high intracellular osmolarity and impaired homeostasis imbalance (**Figure** **6**).^[^ ^70^ ^]^ This process caused ROS increment, caspase‐1 activation, GSDMD cleavage and IL‐1β release. In vivo results showed that ZrNPs‐mediated pyroptosis revealed an excellent antitumor immunity response with DC maturation and memory T cell activation, tumor growth and metastasis inhibition. However, the biocompatibility and biosafety issues of the inorganic nanoparticles in vivo should be paid more attention. Sun et al. constructed a prodrug nanomicelle (AOZN) self‐assembly from γ‐oryzanol (Orz) as a DNA methyltransferases inhibitor and α, β‐methylene adenosine 5′ diphosphate (AMPCP) as an adenosine inhibitor connected with a GSH‐responsive crosslinker (Figure 6).^[^ ^77^ ^]^ The released Orz upregulated GSDMD expression and AMPCP facilitated caspase‐1 activation with ATP level increment, both of which induced tumor pyroptosis. The AOZNs nanoformulation elevated the hydrophobicity of Orz for effective tumor growth inhibition and PD‐L1 responsiveness enhancement. Liang et al. developed a new type of photosensitizer TBD‐3C with membrane targetability and aggregation‐induced emission (AIE) property for PDT mediated pyroptosis‐cancer immunotherapy (Figure 6).^[^ ^68^ ^]^ They claimed that the pyroptotic cells stimulated TAM polarization, DC maturation, CD8^+^ T cell activation and also had the abscopal effect. Tian et al. constructed a hybrid nanoenzyme with GOx encapsulation via biomineralization‐like methodology (Figure 6).^[^ ^88^ ^]^ They found that glycometabolism induced pyroptosis with an antitumor immune response and increased PD‐L1 expression with enhanced ICB sensitivity. They claimed that the nanoparticles combination with anti PD‐L1 inhibited tumor development, increased mice survival, with an immune memory effect for tumor recurrence and metastasis inhibition. Mao et al. found that platinum (II) complexes could serve as photoactivators for the cGAS‐STING pathway activation with mitochondrial/nuclear DNA damage, accompanied by pyroptosis with potent antitumor immune activity.^[^ ^89^ ^]^ Thus, a variety of nanoplatforms can be applied into pyroptosis immunity via osmolarity elevation, ROS generation as well as glycometabolism with GSDMD cleavage during cancer treatment.

### 4.5 Nanomedicines Facilitated GSDME Activation

GSDME is originally identified as DFNA5, that can switch caspase‐3‐mediated apoptosis to pyroptosis.^[^ ^90^ ^]^ The intracellular expression levels of GSDME determine tumor cell death types. When GSDME is highly expressed, it is cleaved by active caspase‐3, and the released N‐terminal domain perforates on the cell membrane, leading to cell swelling, rupture and death for tumor pyroptosis. When GSDME expression level is low, it leads to tumor apoptosis.^[^ ^91^ ^]^ Shao et al. found that GSDME was cleaved by caspase‐3 inducing pyroptosis. The authors also found that GSDME is highly expressed in normal tissues of mice which may cause severe side effects.^[^ ^90^ ^]^ After chemotherapy, the morphology and structure of tissues and organs in GSDME negative mice were intact compared with GSDME positive ones. Therefore, the regulation of GSDME expression is helpful to reduce the toxic and side effects of chemotherapy.

**Figure 7:**
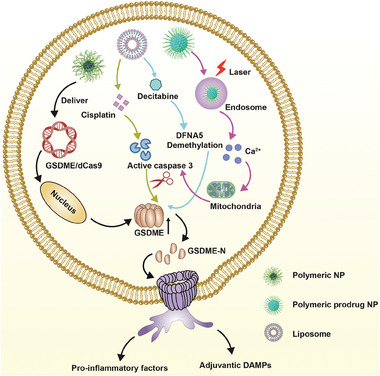
Schematic illustration of nanomedicines mediated GSDME activation for pyroptosis cascade with immunity. Nanocarriers such as liposome, polymeric and prodrug nanoplatform which deliver GSDME/dCas9, cisplatin, decitabine, or generate ROS in endosome with Ca^2+^ overload in mitochondria, elevate GSDME level and facilitate active caspase‐3 formation leading to pro‐inflammatory and DAMPs release.

GSDME‐mediated pyroptosis can be achieved via superoxide radical, calcium ion (Ca^2+^), radiofrequency heat, decitabine and so on, when nanoplatforms are generally constructed for a better antitumor efficacy.^[^ ^67^, ^92^ ^]^ For example, to achieve pyroptosis at different maturation stages of cells, Chen et al. reported an acid‐activated nano‐photosensitizer (ANP) library.^[^ ^93^ ^]^ In short, ANP_EE_ with ultra acid sensitivity at pH 6.5 exhibited a significant phototoxicity at the early endosome (EE) stage. After further investigation, they found that phospholipase C (PLC) specifically localized in the early endosomes, and PDT_EE_ increased inositol 1, 4, 5‐triphosphate (IP3) levels by triggering PLC phosphorylation thereby increasing Ca^2+^ concentration and activating cytochrome C and caspase‐9. After GSDME cleavage by caspase‐3, pyroptosis was induced. This work creatively exploits nanomedicine to regulate pyroptosis activity via targeting to endocytic signaling. To achieve a tumor‐specific pyroptosis, Xu et al. reported a ROS/GSH dual‐responsive prodrug nanomicelle with a high loading of PTX when purpurin 18 (P18) served as a photosensitizer.^[^ ^94^ ^]^ ROS could be generated by P18 under laser irradiation with GSDME‐related tumor pyroptosis and released PTX for chemo‐photodynamic therapy. Gong et al. reported a Nano‐CRISPR scaffold written as Nano‐CD utilizing a specific sgRNA to elevate endogenous GDSME expression, simultaneously release cisplatin for ICD (**Figure** **7**).^[^ ^13d^ ^]^ Nano‐CD revealed an efficient tumor growth inhibition in both primary and recurrent melanomas. This study points out the way that utilize gene editing methodology to induce pyroptosis cascade with antitumor immunity.

Hypermethylation of DFNA5 gene will cause GSDME absence in most tumor cells. Zhang et al. developed a strategy that utilized decitabine (DAC) for the demethylation of DFNA5 thereby triggering pyroptosis, simultaneously administrating nanoliposome loaded with cisplatin (LipoDDP) for caspase‐3 activation.^[^ ^95^ ^]^ In vivo results showed the successful GSDME silence reversal, pyroptosis occurrence enhancement, DC maturation and T cell proliferation increment, anti‐metastasis achievement and recurrence inhibition, which provide a key insight into cancer immunotherapy. Yang et al. developed a bivalent gold nanocluster which induced pyroptosis via Au atom mediated radiofrequency (RF)‐heating effect.^[^ ^96^ ^]^ The results indicated that RF‐mediated pyroptosis elicited tumor ICD with a synergistic antitumor efficacy after combination with decitabine. It also enhanced αPD‐1 immunotherapy efficacy with tumor metastasis inhibition and relapse. Thus, multiple strategies including ROS focusing on early endosome, CRISPR technique, epigenetics application and RF effect can trigger pyroptosis via GSDME cleavage, most of which have huge clinical potentials.

## 5 Autophagy‐Elicited Cancer Immunity

Autophagy is a lysosomal catabolic process that maintains intracellular environment homeostasis by degrading damaged, denatured or senescent proteins and organelles.^[^ ^97^ ^]^

The autophagy process includes induction, assembly and formation of autophagosomes, docking and fusion of autophagosomes and lysosome membrane degradation and reuse of autophagosome.^[^ ^98^ ^]^ Both internal and external stimulation can induce cell autophagy.^[^ ^99^ ^]^ For example, depletion of amino acid and serum are able to induce high autophagy levels. Oxidative stress leads to cell autophagy to remove intracellular damaged proteins and dysfunctional organelles.

In TME, T cells rely on autophagy to support their survival and differentiation. The basal level of autophagy in naive T cells maintains their quiescent state and protects them from ROS mediated apoptosis of mitochondria. High levels of lactic acid in tumors interfered with autophagy of naive T cells and impaired anti‐tumor response in mouse models.^[^ ^1a^ ^]^ Autophagy is critical to the integrity of mitochondria, which determine the differentiation of effector T cells through metabolic reprogramming.^[^ ^100^ ^]^ High level of arginase 2 (ARG2) expression in tumor‐infiltrating Tregs can maintain high level of autophagy. The loss of autophagy in Tregs shifts their metabolism from oxidative phosphorylation to glycolysis with active mammalian target of rapamycin complex 1 (mTORC1) and MYC, resulting in forkhead box protein P3 instability.^[^ ^101^ ^]^ Increased apoptosis and functional deficits of autophagy deficient Tregs contribute to the enhancement of tumor resistance. TAMs degrade apoptotic cancer cells using phagocytosis associated with lipotubule associated protein (LAP) 1A/1B light chain 3 (LC3). LAP defects in TAMs lead to the release of mitochondrial DNA from apoptotic cancer cells, thereby inducing a type I interferon response through the cGAS stimulator (STING) pathway and antitumor activity of interferon genes.^[^ ^13^, ^100^ ^]^

In the process of tumor ICD, autophagy activation accelerates the lysosomal hydrolysis of cytoplasmic components, thus promoting endogenous antigens processing, ATP secretion, immune cell recruitment and infiltration.^[^ ^13^, ^100^ ^]^ However, autophagy plays a dual role in tumor growth.^[^ ^102^ ^]^ It can also generate free fatty acids which are needed for cancer cell survival, leading to chemotherapy resistance and immune evasion. Janji et al. found that autophagy led to granzyme‐B degradation accompanied by tumor lysis alleviation.^[^ ^103^ ^]^ In addition to tumor cell autophagy, immune cells (e.g., DCs, TAMs) can also activate host immune system for a high antitumor immunity by inducing (e.g., DCs) or inhibiting autophagy (e.g., TAMs).^[^ ^104^ ^]^ Therefore, ether autophagy inhibition or excessive activation is able to be used in achieving a high antitumor immune response. Autophagy inducers mainly include rapamycin, STF‐62247, and inhibitors are chloroquine (CQ), ammonium chloride and 3‐methyladenine.^[^ ^98^, ^105^ ^]^ However, all of them have shortcomings such as rapid metabolism, easy excretion and low tumor accumulation. Properly designed nanosystems have long circulation time and can improve tumor accumulation through EPR effect, which partially address the above issues.^[^ ^106^ ^]^ In this part, we will introduce the recent progress of autophagy in tumor immunotherapy based on the inhibition or induction of autophagy mediated by nanosystems.

### 5.1 Nanosystems Based on Autophagy Inhibitors

**Figure 8:**
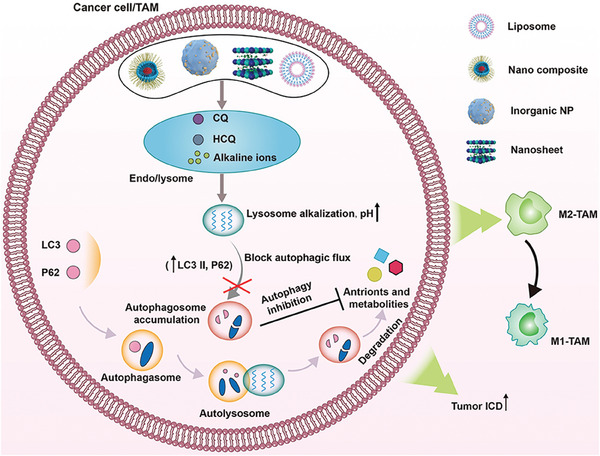
Schematic illustration of nanomedicines mediated autophagy inhibition in cancer cell or TAMs for immune modulation.

In consideration that multiple therapies are accompanied by autophagy with antitumor resistance, autophagy inhibition may be a promising strategy in strengthening antitumor immune response. In order to resolve the problems of small autophagy inhibitors, Yu et al. built a semiconductor polymer nanocomposite (SPN_CN_) by loading the autophagy inhibitor CQ and IDO inhibitor NLG919 into a singlet oxygen (^1^O_2_) responsive semiconductor polymer nanoparticle (SPN) (**Figure** **8**).^[^ ^107^ ^]^ Under laser irradiation, SPN_CN_ produced ^1^O_2_ to induce ICD, and destroyed ROS sensitive bond to achieve the precisely intracellular release of CQ and NLG919. CQ inhibited tumor autophagy, and amplified PDT and ICD with DC maturation. NLG919 interfered with immunosuppressive tryptophan (Trp) metabolism, elicited an effector T cell activation and inhibited Treg proliferation. In vivo results indicated that SPN_CN_ prolonged survival rate, inhibited tumor growth without systemic toxicity.

TAMs autophagy inhibition is an effective way to strengthen antitumor immunoefficacy. For example, Chen et al. constructed a hollow mesoporous Prussian blue (HMPB) nanosystem (Man‐HMPB/HCQ) with mannose (Man) modification and hydroxychloroquine (HCQ) adsorption.^[^ ^108^ ^]^ They found that the nanosystem induced TAM polarization through interferon regulatory factor 5 pathway, suppressed TAMs autophagy via HCQ. Macrophage and thylakoid (TK) hybrid membrane was coated on Man‐HMPB/HCQ surface (TK‐M@Man‐HMPB/HCQ) to reduce reticuloendothelial system uptake in vivo, promote tumor tissue enrichment and relieve hypoxia. This report subtly utilizes mimetic hybrid nanosystem to alleviate hypoxia, induce TAMs polarization with autophagy inhibition, providing a novel strategy for TME modulation. In another example, Gao et al. reported polyethylene glycol‐conjugated gold nanoparticles, named as PEG‐AuNPs, which blocked TAM autophagy via lysosome alkalization for membrane permeabilization, followed by M2‐like TAM polarization inhibition and antitumor efficacy enhancement (Figure 8).^[^ ^109^ ^]^ In this research, nanomaterials’ biological effects are found for TAMs polarization modulation, providing a new insight into exploiting nanomaterials’ property for effective cancer therapy. To resolve tumor ICD mediated autophagy, Wang et al. constructed two liposomal nanosystems separately with an ICD inducer (LipSHK) (Shikonin, SHK) and HCQ/ATP (LipHCQa) encapsulation for autophagy inhibition (Figure 8).^[^ ^110^ ^]^ They found that the combination of LipSHK and LipHCQa maximized the ICD efficacy for a robust antitumor immunity and emerged a clinical translation potential. To reverse immunosuppressive TME, Liu et al. reported layered double hydroxide nanoparticles (LDH NPs) with weakly alkaline property that could neutralize acid with MDSC, Treg, M2‐TAMs inhibition, and block tumor cell autophagy for a robust cancer immunotherapy efficacy.^[^ ^111^ ^]^ Thus, TME modulation can be effectively combined with tumor autophagy inhibition for a synergistic effect. However, the immune adjuvant function of LDH NPs as well as their ability to inhibit TAMs autophagy aren't mentioned in this study, both of which may play key roles in elicit host immunity.

### 5.2 Nanosystems Based on Autophagy Inducers

#### 5.2.1 Small Molecule Inducers

Autophagy levels determine their antitumor or protumor properties. In fact, there is a critical point for autophagy activation. When the autophagy level is below the critical point, it contributes to tumor growth by removing damaged cell components. When the autophagy level is above the critical point called as “excessively activated autophagy”, it will lose the function to protect tumor cells and cause tumor cell death by triggering autophagy death pathway. STF‐62247 is a small molecule autophagy inducer, generally used in cancer therapy. For example, Wang et al. developed an on‐demand autophagy cascade amplification nanoparticle (ASN) to separately promote oxaliplatin (OXA) mediated ICD and STF‐62247 mediated autophagy for enhanced antitumor immunoefficacy.^[^ ^112^ ^]^ The self‐assembled autophagy sensitive micelles (C‐TFG) were electrostatically bound to the OXA grafted hyaluronic acid (HA) prodrug (HA‐OXA) to obtain ASN. After internalization, HA‐OXA was first hydrolyzed by hyaluronidase. In the intracellular high reduction condition, the bounded OXA was reduced to free OXA, leading to tumor ICD, stimulating autophagy from the static state to mild and activated phase. Then, C‐TFG micelles immediately disassociated at the stimulation of oxaliplatin mediated autophagy accompanied by STF‐62247 release. This process accurately transformed autophagy into an “over activated” state, leading to tumor autophagy death with robust tumor antigen processing capacity.

#### 5.2.2 Polypeptide Autophagy Inducer

**Figure 9:**
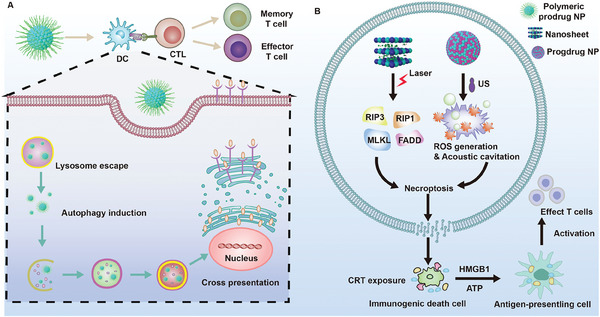
A) Schematic illustration of polymeric prodrug nanoplatform mediated autophagy enhancement in DC for CTL activation. B) Schematic illustration of nanomedicines mediated necroptosis cascade with immunity response.

As a type of innate immune cells, DCs play important roles in activating host immune response. Autophagy activation of DCs will promote DC maturation and antigen cross‐presentation, and generate antigen‐specific T cells to eliminate tumors.^[^ ^104^ ^]^ For example, Wang et al. synthesized pH responsive polymers through Michael addition reaction, and then effectively connected the autophagy inducing peptide (Beclin1) and antigen peptide ovalbumin (OVA) to the polymer skeleton, and formed nanoparticles (NP‐B‐OVA) (**Figure** **9**A).^[^ ^113^ ^]^ NP‐B‐OVA was uptaken by DCs through endocytosis that could escape from lysosomes with antigen release. Because of the pH responsiveness of the graft copolymer, this lysosomal dependent process may contribute to the decomposition of nanoparticles and the exposure of Beclin1. By observing the co‐localization of NP‐B‐OVA‐Cy5 and autophagic marker (GFP‐LC3), it was further proved that NP‐B‐OVA induced autophagy by the formation of autophagosomes. In vivo results showed that DC autophagy significantly enhanced effector T cell activation, inhibited tumor growth and prolonged the median survival of mice.

## 6 Necroptosis

Necroptosis is a programmed lysate cell death pathway that is thought to play a role in killing pathogen‐infected and/or damaged cells during certain degenerative or inflammatory diseases.^[^ ^114^ ^]^ It can be induced by a variety of innate immune signaling pathways, including those triggered by stimulation of RIG‐I receptors, Toll‐like receptors (TLRs) and death receptors. As a part of complex IIa, caspase‐8 plays a central role in necroptosis can be activated and induce cancer cell apoptosis through the cleavage of downstream molecules. When caspase‐8 is inhibited, receptor interaction of protein kinase 1 (RIPK1) and RIPK3 can form a necrotizing complex with receptor‐interacting protein kinase homotypic interaction motif (RHIM) through isotypic interactions, thereby activating mixed lineage kinase domain like (MLKL) protein via phosphorylation. Typical death receptor‐mediated apoptosis pathways include death domain receptors (e.g., tumor necrosis factor (TNF) receptors, Fas and TLR3/4 receptors) triggering RIPK1‐RIPK3‐MLKL. Active RIPK1 is recruited in an oligomer complex that includes Fas‐associating protein with a novel death domain (FADD), caspase‐8 and caspase‐10. In the absence of caspase‐8, RIPK1 recruits and phosphorylates RIPK3 and re‐phosphorylates MLKL to form oligomers. MLKL oligomers are then transported to phosphatidyl inositol phosphate (PIP)‐rich plaques in the plasma membrane, forming large pores that cause necroptosis by allowing ion influx, cell swelling and membrane lysis, and the release of intracellular contents. Necroptosis is heavily dependent on RIPK3 and MLKL, and activation of MLKL is defined as a characteristic feature of necroptosis. In addition to the classical pathway, the immune system has also evolved to bypass RIPK1‐mediated necrosis, the upstream signal of RIPK3‐MLKL. Proteins containing the RHIM domain, such as Z‐DNA binding protein 1 (ZBP1), and adaptors containing the tir domain can induce IFN‐β to bind directly to RIPK3 via its RHIM domain, inducing necrotic activation independent of RIPK1.^[^ ^115^ ^]^ Nowadays, necroptosis has been developed as a cell death pathway cascade with cancer immunotherapy to achieve a high antitumor activity.^[^ ^116^ ^]^

Effectors in necrotic apoptosis such as RIPK1 and RIPK3 can directly regulate the function of immune cells, independent of cell death. As has been reported, RIPK3‐mediated phosphoglycerate mutase 5 (PGAM5) activation promoted a NK T‐cell‐mediated antitumor immune response by activating nuclear factor of activated T cells (NFAT) and dephosphorylation of dynamically‐associated protein 1 (Drp1) in a process independent of the necrotic pathway.^[^ ^1a^ ^]^ In addition, in isogenic melanoma and lung adenocarcinoma models, the injection of necrotic tumor cells activated by RIPK3 into existing tumors enhanced antitumor immunity.

### 6.1 Nanomedicine‐Based RIPK3‐MLKL Dependent Necroptosis

The classical RIPK3‐MLKL pathway mediated necroptosis assisted by nanotechnology has been widely investigated. Liu et al. constructed BPCP nanoplatform composed of BP, bPEI‐PEG and CpG by inducing cell necrosis through photothermal therapy (PTT) where PEG‐bPEI was connected to BP by non‐covalent bond and the immune adjuvant CpG was connected by electrostatic interaction (Figure 9B).^[^ ^117^ ^]^ The expressions of RIPK1 and RIPK3 were significantly increased in 4T1 cells after irradiation, and the downstream of mitochondrial membrane permeability and production of ROS were also observed. In vitro results demonstrated that the immunogenicity of BPTT‐mediated cell necroptosis was sufficient to trigger immunotherapeutic effects on cancer cells. After irradiation of the primary tumor, notable tumor growth inhibition was found in the contralateral one, suggesting that photothermal ablation can activate a systemic immune response. The expression of IL‐2, TNF‐α, IFN‐γ increased in BPCP plus laser group, proving that BPTT activates the immune response and alleviates the immunosuppressive tumor microenvironment.

TNF‐α can induce tumor cell necroptosis while the direct intravenous administration may lead to systemic toxicity. To cope with the issue, Zhou et al. designed a liposomal nanoplatform with TNF‐α encapsulation which notably induced HMGB1 release.^[^ ^118^ ^]^ In vivo results showed that elevated CD8^+^ T cells, notable necroptosis, high IFN‐γ and IL‐6 levels were observed in mice treated with liposome‐TNF‐α plus anti PD‐L1. Chemodynamic therapy (CDT) and PTT can also induce necroptosis.^[^ ^119^ ^]^ Wei et al. constructed nanoflowers with 2‐glucose oxidase (DG) and GOx modification on the surface of rose‐like MoS_2_ in sequence (MPGGFs).^[^ ^120^ ^]^ Due to the inherent and excellent photothermal conversion efficiency of MoS_2_ nanoflowers (MF), MPGGFs effectively induced tumor necroptosis, which directly triggered the host immunity through of antigen‐specific T cell activation. The released GOx consumed glucose and oxygen in an enzyme‐catalyzed biochemical reaction, which then produced large amounts of H_2_O_2_ in situ. Exogenous and endogenous H_2_O_2_ were further catalyzed by the exposed MF to form highly toxic •OH through oxidase‐like activity, leading to efficient, self‐amplifying tumor necroptosis. Simultaneously, the photothermal conversion characteristics of MF further generated local high temperature under irradiation, and accelerated tumor necroptosis. Thus, necroptosis induced by PTT, CDT as well as TNF‐α depending on RIPK3‐MLKL pathway can be utilized in strengthening antitumor immune response via nanotechnology strategies.

### 6.2 Nanomedicine‐Based RIPK3‐MLKL Independent Necroptosis

In addition to the above nanosystems that stimulate systemic immunity based on the conventional necroptosis pathway, Park et al. reported a polymeric nanobubble (NB) that independent of RIPK3/MLKL pathway induced necrosis via the acoustic cavitation effect (Figure 9B).^[^ ^121^ ^]^ NBs were prepared by oil‐in‐water emulsion method, in which perfluoropentane (PFP) acted as the gas precursor, and amphiphilic polymer conjugate served as the reservoir. The conjugate was composed of pegylated carboxymethyl dextran (PEG‐CMD) as a hydrophilic backbone and chlorin e6 (Ce6) as a hydrophobic sound sensitizer to produce ROS. Although NB produced the lowest amount of ROS, its cytotoxicity was comparable to that of Ce6, suggesting that the cytotoxicity of NBs was significantly enhanced by acoustic cavitation induced necroptosis. Regardless of the expression of RIPK3 and MLKL, tumor cells showed necrotic morphological characteristics when treated with NB under US irradiation, suggesting that the occurrence of cell necroptosis was independent of RIPK3/MLKL. Compared with other controls, CD86 expression on DC surface significantly increased in US plus NB group, when the prominent tumor regression may attribute to ROS‐mediated cell death and bubble‐induced necroptosis. The combination of NB with US and aPD‐L1 significantly enhanced the antitumor response in both primary and metastatic tumors.

When exposed to low‐dose chemotherapy, radiotherapy, or extreme physicochemical stress, cancer cells will die and induce strong host antitumor responses by releasing subcellular components leading to cancer cell membrane immunogenicity. Thus, can the direct use of cancer cell membrane cause tumor necroptosis? The answer is yes. Chen et al. designed nanosized “artificial necrotic cancer cells” to trigger an immune response.^[^ ^122^ ^]^ The “artificial necrotic cancer cell” (AHSP70p‐CM‐CaP) was consisted of a phospholipid bilayer and phosphate calcium nuclei that co‐delivered cancer membrane protein (CM), α‐helix HSP70 peptide (αHSP70p), and CpG to NK cells and APCs. Effective lymph node transportation and T cell response were observed in mice with αHSP70p‐CM‐CaP vaccine treatment. Importantly, αHSP70p‐CM‐CaP also induced an increase of IFN‐γ^+^ CD8^+^ T cells. When treated with anti‐PD‐1 in a B16OVA melanoma model, αHSP70p‐CM‐CaP resulted in a target cell death, tumor regression and a long‐term immune memory effect in vivo. The design about the “artificial necrotic cancer cell” greatly broadens the necroptosis application in cancer treatment and host immunity evocation.

## 7 Apoptosis Cascade with Cancer Immunotherapy

Apoptosis refers to the active and physiological cell death process under certain conditions which is controlled by the internal gene.^[^ ^2^, ^123^ ^]^ There are obviously morphological characteristic changes after apoptosis, such as cell shrinkage, plasma membrane outflow bubbles.^[^ ^2c^ ^]^ Meanwhile, apoptosis is also accompanied by typical features including chromosomal DNA fragmentation into nucleosomal units, phosphatidylserine (PtdSer) exposure on cell surface and mitochondrial potential loss. Apoptosis can be generally divided into intrinsic and extrinsic pathways.^[^ ^2c^ ^]^ The intrinsic apoptosis pathway refers to mitochondrial outer membrane permeability increment accompanied by cytochrome C release into the cytoplasm, which further promotes apoptosome formation and caspase‐3/6/7 activation.^[^ ^124^ ^]^ This pathway also known as mitochondrial pathway is controlled by B‐cell lymphoma‐2 (BCL2) family members. The extrinsic apoptosis pathway is mainly initiated by cell surface death receptors including tumor necrosis factor receptor 1/2, Fas and tumor factor related apoptosis inducing ligand (TRAIL) receptors DR4 and DR5.^[^ ^125^ ^]^ When activated by their ligands, the receptors are induced to form trimers and aggregate in cell membrane, which subsequently recruit adaptor proteins with caspase‐8/10 activation and death signal complex formation. In this section, we mainly introduce nanomedicines mediated intrinsic or extrinsic apoptosis pathway.

### 7.1 Nanomedicines Mediated Intrinsic Apoptosis Pathway

Nanotechnology‐assisted apoptosis such as sonodynamic therapy (SDT), PDT, gene therapy, protein therapy and chemotherapy, has achieved huge advances.^[^ ^126^ ^]^ For example, during SDT, ultrasound can activate sonosensitizers leading to endogenous apoptosis related factor (e.g., Bax, caspase‐9, caspase‐3) increment and cell membrane potential alteration.^[^ ^127^ ^]^ However, due to the anti‐apoptosis mechanism, the antitumor efficacy mediated by SDT alone is insufficient, which needs the combination therapy with other therapeutic approaches.

**Figure 10:**
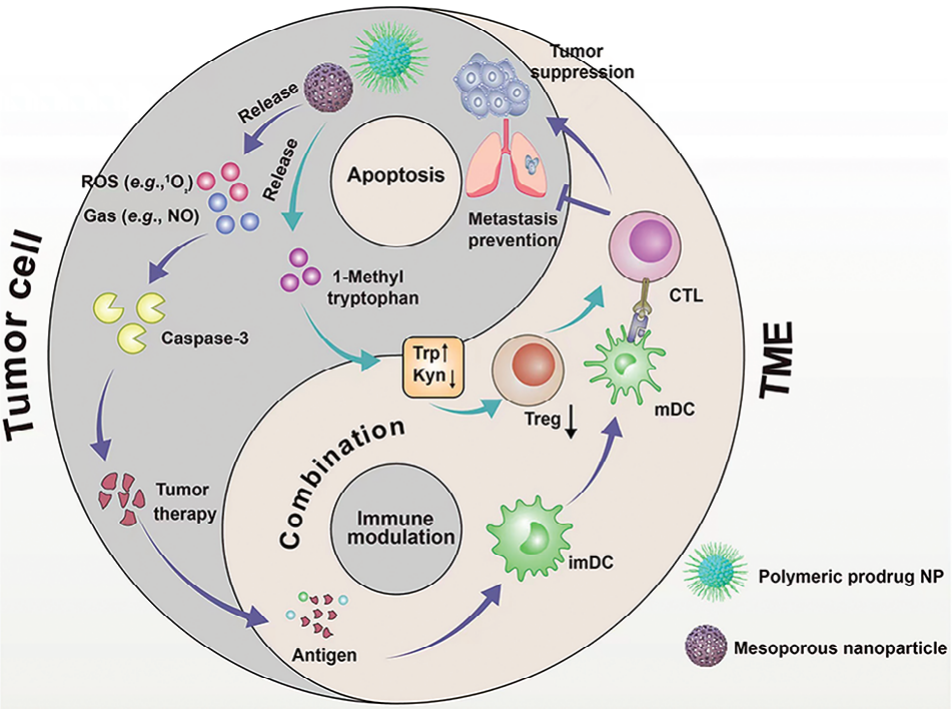
Schematic illustration of nanomedicines mediated intrinsic apoptosis and combination with TME modulation to activate host immune response.

Inspired by the synergistic antitumor effect of ferroptosis and apoptosis, Zhou et al. constructed a liposomal nanocomposite system simultaneously encapsulated with the clinically approved iron supplement ferumoxytol and a nanosonosensitizer (PpIX).^[^ ^128^ ^]^ The experimental results showed an up‐regulated expression of pro‐apoptotic markers (e.g., caspase 3, Bax), indicating that ferumoxytol and SDT synergistically improved the apoptotic index and the therapeutic efficacy. To enhance antitumor efficacy and inhibit tumor metastasis, Wang et al. prepared a multifunctional nanovaccine with L‐arginine (LA) encapsulation into black mesoporous titanium dioxide (BMT) (**Figure** **10**).^[^ ^129^ ^]^ In this system, LA was used as an exogenous nitrogen oxide (NO) supplement for gas therapy and BMT as a sonosensitizer for SDT. Under ultrasound stimulation, BMT and LA produced ^1^O_2_ and NO, respectively. In addition, ^1^O_2_ generated by BMT could promote the oxidation of LA for more NO generation. The elevated intracellular ^1^O_2_ and NO levels caused a strong oxidative stress and DNA double strand cleavage, both of which eventually induced cell apoptosis. After combination with anti‐PD‐L1, the nanosystem blocked immune checkpoint, restored T cell activity, enhanced antitumor immune responses, and inhibited tumor metastasis.

About PDT mediated apoptosis, Wu et al. synthesized a light triggered coumarin derivative exploiting the biological precursor 5‐aminolevulinic acid (5‐ALA) conjugation to coumarin.^[^ ^130^ ^]^ Both the coumarin derivative and mitochondrial targeting compound triphenylphosphine (TPP) were conjugated on carbon dots (CDS) through covalent bond to form nanosystem (CD‐ALA‐TPP). Conjugated TPP improved cell penetrating ability as well as delivered 5‐ALA into the mitochondria. Upon biphoton irradiation, this nanosystem released 5‐ALA molecules, which were metabolized in mitochondria via a series of biosynthesis to generate protoporphyrin IX (PpIX). Under near infrared (NIR) irradiation, PpIX produced ^1^O_2_ resulting in mitochondrial oxidative damage for further cell apoptosis. To resolve the problems of tumor heterogeneity and immunosuppressive TME, Song et al. used a caspase sensitive peptide Asp‐Glu‐Val‐Asp (DEVD) to bond the photosensitizer PpIX and immune checkpoint inhibitor 1‐methyl tryptophan (1MT) to obtain chimeric PpIX‐1MT for PDT evoked apoptosis and TME modulation (Figure 10).^[^ ^131^ ^]^ Under NIR irradiation, PpIX‐1MT nanoparticles generated ROS leading to caspase‐3 expression and tumor antigen production for cancer cell apoptosis and host immune response provocation. Subsequently, caspase‐3 cleaved sensitive peptide DEVD leading to 1MT release from nanoparticles, which reversed IDO‐mediated immunosuppression and activated CD8^+^ T cells. This cascade synergistic effect amplified apoptosis efficacy and inhibited primary tumor growth and lung tumor metastasis, providing a solution to resolve clinical tumor recurrence and metastasis. In another example, Pu et al. developed a semiconducting polymer (SP) nano‐regulator (SPN_T_) conjugation with 1‐methyltryptophan (M‐Trp) via apoptosis‐cleavable linker, which was cleaved under laser irradiation for PDT mediated immunogenic apoptosis (Figure 10).^[^ ^132^ ^]^ The released M‐Trp inhibited IDO activity, reduced Tregs accompanied by enhanced cytotoxic T lymphocytes infiltration. Thereby, SDT, PDT as well as gas therapy are able to cause tumor apoptosis, which can combine with immunomodulator for new strategies in exerting immunoefficacy.

### 7.2 Nanomedicines Mediated Extrinsic Apoptosis Pathway

Gene therapy means to change the biological properties of cell to achieve a high therapeutic efficacy through target gene controlling or pathogenic gene replacement. It can fundamentally control disease progression by directly repressing the expression of pathogenic genes.^[^ ^133^ ^]^ MDM2 is a negative regulator of tumor suppressor gene p53.^[^ ^134^ ^]^ Thus, blocking the interaction between MDM2 and p53 can activate p53 pathway, leading to cancer cell cycle arrest and apoptosis.^[^ ^135^ ^]^ Yu et al. designed a novel pH responsive composite nanoparticle via siRNA‐MDM2 delivery for non‐small cell lung cancer (NSCLC) H2009 treatment.^[^ ^136^ ^]^ The results showed that knockdown of MDM2 in H2009 cells led to p21 up‐regulation, caspase‐3 pathway activation and apoptosis inducement with high antitumor activity.

To improve the stability and efficacy of therapeutic RNAs, Duan et al. incorporated RNA delivering lipid nanoparticles (HLP) into a photosensitive hydrogel (GM scaffold) to form G‐HLP/RNAs system. This system used STAT3 siRNA and BIM mRNA as therapeutic RNAs to induce C26 cell apoptosis. Under irradiation, injected GM scaffold promoted macrophage infiltration, DC maturation and T cell activation, while the therapeutic RNA induced tumor cell apoptosis. The G‐HLP/RNAs system effectively inhibited C26 tumor growth with 71.9% tumor weight inhibition. G‐HLP/RNAs is expected to be a valuable tool for tumor gene therapy due to the ability of controlled release of RNA, immune cell recruitment and tumor apoptosis inducement.

**Figure 11:**
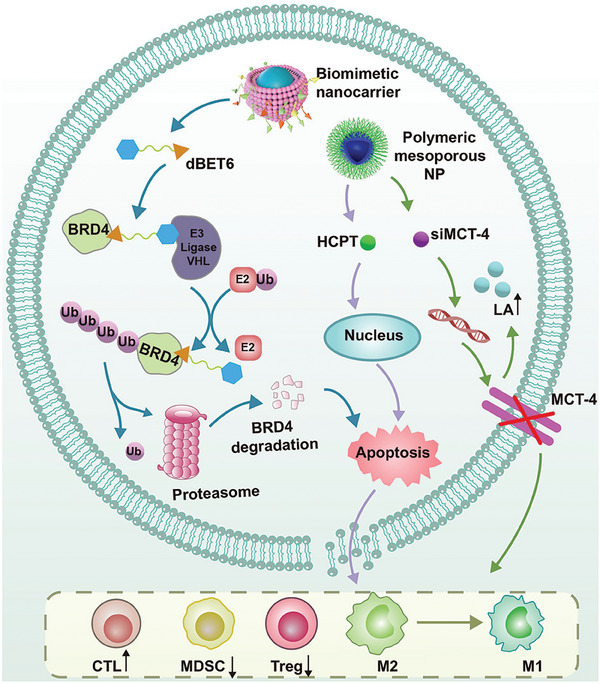
Schematic illustration of nanomedicines mediated extrinsic apoptosis for antitumor immunity.

A variety of proteins, such as Bax, Bak, p53, Smac and TRAIL, are capable of inducing tumor cell apoptosis.^[^ ^137^ ^]^ Among of them, TRAIL is a promising therapeutic protein, which selectively induces tumor cell apoptosis after binding to its cognate death receptors DR4 or DR5 expressed on tumor cell surface.^[^ ^138^ ^]^ For example, Liu et al. used fibroblast cell membranes which expressed TRAIL, to coat CQ nanoparticles to form biomimetic nanosystem (TM‐CQ/NPs) (**Figure** **11**).^[^ ^139^ ^]^ This nanosystem effectively evaded from macrophage clearance with an enhanced tumor targetability. In addition, this fibroblast membrane specifically induced tumor cell apoptosis via TRAIL interaction with its cognate death receptor simultaneously overcoming TRAIL delivery barrier. This nanosystem showed significant tumor growth inhibition and tumor cell apoptosis rates in both orthotopic liver and peritoneum tumor models.

Bromodomain protein 4 (BRD4) is a member of the bromodomain and extra terminal (BET) protein family that primarily regulates genes involved in apoptotic functions.^[^ ^140^ ^]^ Recent studies have shown that degradation products of BRD4 (e.g., dBET6) are able to efficiently induce apoptosis in human lung cancer cells. However, dBET6 has poor solubility and low bioavailability, which severely affect their therapeutic efficacy.^[^ ^141^ ^]^ Proteolysis targeting chimeras (PROTAC) have emerged as a promising therapeutic strategy with the ability to precisely degrade target proteins. Zhang et al. developed a novel multifunctional nano PROTAC (named as CREATE) to precisely degrade BRD4.^[^ ^142^ ^]^ Specifically, the authors used a pH/GSH responsive polymer (disulfide linked poly (lactic‐*co*‐glycolic acid), referred to DS‐PLGA) to load dBET6 self‐assembly into nanoparticles, which was coated by an engineered lung cancer cell membrane (CRV‐LLCM) with dual targeting ability to form a biomimetic nanosystem (Figure 11). CREATE can be targeted to both cancer cells and TAMs. The pH/GSH responsiveness of the nanosystem promoted intracellular release of dBET6, leading to BRD4 degradation, lung cancer cells and TAMs apoptosis, TME modulation, and subcutaneous and orthotopic lung tumor growth inhibition.

Chemical drugs such as cisplatin, arsenic trioxide and hydroxycamptothecin (HCPT) can induce tumor cell apoptosis through endogenous pathways.^[^ ^143^ ^]^ Free camptothecin (CPT) has notable disadvantages such as highly toxic to normal cells, low tumor accumulation and multiple drug resistance (MDR). It is essential to resolve the above issues via nanotechnology.^[^ ^55^, ^144^ ^]^ Yang et al. constructed a charge reversal nanosystem that self‐assembled from pH/ROS cascade responsive polymer CPT prodrug with β‐Lapachone encapsulation.^[^ ^145^ ^]^ In the weakly acidic TME, the surface charge of this micellar system can be changed from negative to positive, thereby enhancing tumor cell internalization. Intracellular release of β‐Lapachone produced more ROS under the catalysis of NADPH quinone oxygen reductase 1 (NQO1), further leading to the self‐amplification and disintegration of micelles and drug release. ROS production by β‐Lapachone was accompanied by NAD(P)H/ATP consumption and P‐glycoprotein (P‐gp) down‐regulation, which inhibited drug efflux and overcame multidrug resistance. In addition, excessive ROS production by β‐Lapachone cooperated with CPT to further promote tumor cell apoptosis. In vivo and in vitro studies showed that the combination of pH responsive charge inversion and ROS level up‐regulation achieved a potent antitumor activity.

The intracellular highly active glycolysis promotes excessive lactic acid accumulation in TME leading to an immunosuppressive microenvironment. Li et al. constructed polyethylene glycol modified GSH responsive hollow mesoporous organic silica (HMON) nanoplatform with HCPT and monocarboxylic acid transporter‐4 siRNA (siMCT‐4) encapsulation to form HMON@HCPT‐BSA‐PEI‐CDM‐PEG@siMCT‐4.^[^ ^146^ ^]^ This nanoplatform dissociated in the weakly acidic TME and intracellular high GSH level with the continuous release of HCPT and siMCT‐4, resulting in the intracellular lactic acid increment as well as tumor cell apoptosis. In addition, the extracellular lactic acid decrement facilitated the transformation of TAMs from M2 phenotype into M1, enhanced the activity and infiltration of CD8^+^ T cells in vivo. This cascade responsive nanoplatform improved cell apoptosis efficiency, regulated the immunosuppressive TME, and inhibited lung metastases of B16F10 melanoma and 4T1 breast cancer.

## 8 Cuproptosis

Copper is an important mineral nutrient, widely involved in cell proliferation and death pathways. Recently, Tsvetkov et al. found an unexpected mechanism when copper could target to mitochondria triggering a new type cell death named as cuproptosis.^[^ ^147^ ^]^ Cuproptosis mainly depending on mitochondrial respiration is different from the known death mechanism. It occurs through the direct binding of copper to the lipid component during the tricarboxylic acid (TCA) cycle. This process leads to an aggregation of lipid proteins and the loss of iron‐sulfur tuftsin, which results in proteotoxic stress and ultimately cell death. It is also reported that cuproptosis may happen by p53 via regulating the biogenesis of iron‐sulfur clusters and GSH, inhibiting glucose uptake and glycolysis.^[^ ^148^ ^]^ Cuproptosis as a newly discovered copper‐dependent cell death pattern provides a new way to exploring copper‐based nanomaterials for cancer treatment.

### 8.1 Nanomedicines Mediated Cuproptosis

Tsvetkov et al. found that cells mainly relying on mitochondrial respiration were highly sensitive to cuproptosis, nearly 1000 times higher than that depending on glycolysis.^[^ ^147^ ^]^ Cell death was mitigated when using electron transport chain complex, I and II inhibitors and mitochondrial pyruvate uptake inhibitors. In a nanotechnology‐assisted platform, Pan et al. developed a non‐porous copper (I) 1, 2, 4‐triazolic acid ([Cu(tz)]) coordination polymer (CP) nanoplatform for GOx engineering, named as GOx@[Cu(tz)], which was used for a combination therapy of starvation, cuproptosis and PDT in bladder cancer.^[^ ^149^ ^]^ The catalytic activity of GOx was shielded in a non‐porous scaffold, which was not until stimulating by intracellular GSH that glucose consumption could be “turned on” for cancer starvation therapy. Both glucose and glutathione consumption increased the sensitivity of cancer cells to GOx@[Cu(tz)]‐mediated cuproptosis, causing lipid mitochondrial proteins aggregation.

Ferredoxin 1 (FDX1) is a new lipid effector factor, a key regulator of cupric ion mediated cell death and an upstream regulator of protein lipidation. It contributes to the accumulation of toxic lipidized dihydrolipoamide acetyltransferase (DLAT) for subsequent cuproptosis, while FDX1 knockout can lead to the complete loss of protein lipid acylation. The mitochondrial TCA cycle is associated with DLAT, which is a subunit of pyruvate dehydrogenase complex. Copper can directly bind to DLAT and promote disulfide bond dependent aggregation of lipid DLAT. Suo et al. proposed a novel copper/iron hybrid hollow amorphous metal‐organic skeleton (HaMOF), which could act as an oxidative stress amplifier and metabolic disruptor for the synergistic anticancer treatment of cuproptosis/ferroptosis/apoptosis.^[^ ^3e^ ^]^ HaMOF was prepared by combination of unsaturated coordination and etching approach when Cu^2+^, 3, 3′‐dithiobismuth (hydrazine propionate) and Fe^3+^ acted as raw materials. After doxorubicin encapsulation, the surface of HAMOF was decorated with hyaluronic acid. DOX@Fe/CuTH displayed catalytic therapeutic properties, which mediated cellular oxidative stress was amplified by H_2_O_2_ generation and GSH consumption. In addition, the nanoplatform caused mitochondrial dysfunction, copper transporter ATP7A and iron transporter FPN 1 down‐regulation, resulting in metabolic disorders and copper/iron retention in the cytoplasm to produce •OH. Simultaneously, Cu^2+^ overload induced DLAT accumulation for cuproptosis. The results showed that DOX@Fe/CuTH had a good tumor inhibition ability without obvious side effects. Xu et al. reported a smart nanoplatform (named as Au@MSN‐Cu/PEG/DSF) for photothermally triggered drug release cascade with cuproptosis and apoptosis.^[^ ^150^ ^]^ The released DSF chelated with Cu^2+^ in situ to generate a highly cytotoxic bis(diethyldithiocarbamate)‐copper (CuET) causing cell apoptosis and formed Cu^+^ promoting toxic mitochondrial protein aggregation, leading to cuproptosis. Synergistic with PTT, Au@MSN‐Cu/PEG/DSF effectively killed tumor cells and inhibited tumor growth. These results provide a promising perspective and inspire the design of advanced nanotherapeutic platforms for potential cancer treatment based on cuproptosis. The total DLAT protein expression decreased in Au@MSN‐Cu/PEG and Au@MSN‐Cu/PEG/DSF treated groups, indicating that Cu^+^ binding to DLAT caused DLAT oligomerization followed by proteotoxic stress and cell death.

### 8.2 Nanomedicines Mediated Cuproptosis Cascade with Cancer Immunotherapy

**Figure 12:**
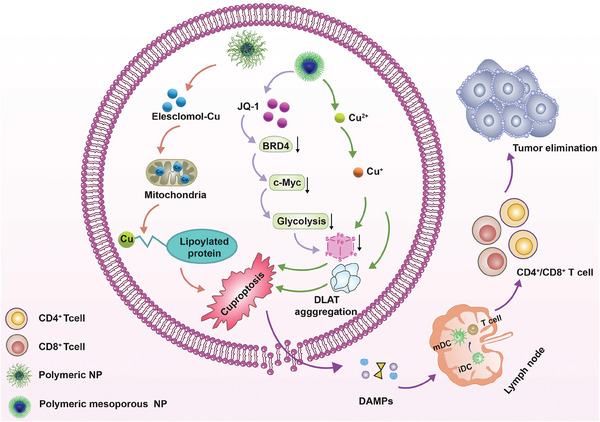
Schematic illustration of nanomedicines mediated cuproptosis cascade with antitumor immunity.

Whether cuproptosis has an intrinsic relationship with cancer immunotherapy is an interesting point. Wang et al. analyzed the TIMER database and showed FDX1 as the key regulator of cuproptosis, the expression of which was positively correlated with CD8^+^ T cells, NK cells and neutrophils infiltration, and negatively correlated with CD4^+^ T cells and CAFs levels. Flow cytometer was executed to detect CD4^+^ and CD8^+^ T cell immunoinfiltration levels in colon adenocarcinoma (COAD) patients with high FDX1 expression, results of which were completely consistent with TIMER database analysis. In another report, Xing et al. constructed a ROS‐responsive polymeric nanoplatform with elesclomol (ES) and Cu co‐encapsulation (NP@ESCu) (**Figure** **12**).^[^ ^14^ ^]^ In the high intracellular ROS levels, the cleavage of ROS bond led to ES/Cu complex release accompanied by selectively targeting to mitochondria for cuproptosis. In addition, the efflux of ES could facilitate the chelation and transportation of extracellular Cu^2+^ into cancer cell. This process caused continuous copper accumulation for robust cuproptosis and the up‐regulated PD‐L1 potentiated immune checkpoint blockade efficacy. This study provides a new way to strengthening immunotherapy efficacy via the combination of cuproptosis and anti‐PD‐L1. Zhang et al. developed a multifunctional immunotherapy nanoreactor (CCJD‐FA) based on cuproptosis via CaO_2_ and JQ1 (bromodomain‐containing protein 4 inhibitor) encapsulation in copper‐based shell decorated with DSPE‐PEG‐FA (Figure 12). They found the nanosystem disassembly with CaO_2_ release in the high intracellular GSH level that elicited H_2_O_2_ generation triggering Fenton reaction with Cu^2+^. The generated O_2_ relived hypoxia and the product Cu^+^ induced cuproptosis when obvious DLAT aggregation was observed after CCJD‐FA treatment accompanied by CRT exposure for DC maturation. This study subtly utilizes cuproptosis combination with metabolism regulation, immune checkpoint blockade and TME modulation, providing a new approach to potentiate antitumor immune response.

## 9 Conclusions and Outlook

Cancer immunotherapy emerges huge advantages over the traditional therapeutic treatments in cancer. Approaches focusing on PCD routes cascade with host immune response activation provide new thoughts into cancer treatment. In this review, we first introduced immune cell types including innate/adaptive immune cells and immunosuppressive cells to support a comprehensive understanding of immune systems. Individual cell death pathway (e.g., ferroptosis, pyroptosis, autophagy, necroptosis, apoptosis and cuproptosis) was separately described including cell death mechanisms and advances in nanotechnology‐assisted pathway. Nanomedicine‐mediated ferroptosis and pyroptosis cancer immunities are mostly explored, probably attributing to the multiple mechanisms in cell death inducement suitable for diverse designs, as well as the diversities of nanomaterials to choose. Although nanotechnology‐based cancer immunotherapy focusing on PCD routes has revealed great achievements, there are still some challenges.

First, the clinical translation of nanomedicine mediated PCD with host antitumor immunity provocation faces to severe obstacles due to the huge differences between rodents and humans, as well as the biosafety issues of nanomaterials. The insurmountable gap of species may increase the difficulty that nanomedicine accumulates in tumor tissue via EPR effect in human. Thus, it is necessary to construct active targeting ligands decorated nanomedicines to improve tumor accumulation via receptor‐mediated internalization during PCD cascaded with antitumor immunity. In addition, some nanomaterials require complicated syntheses which may lead to organic solvents residue resulting in biosafety issues and increase the difficulty to amplify production in an industrial scale for clinical application. Moreover, the high dosage of metal‐based nanomaterials during ferroptosis, pyroptosis, autophagy and cuproptosis may bring in metal ion‐based toxicity in vivo. Thus, nanomaterials with targeting modification, biodegradable property, feasible synthesis as well as relatively low dosages are critical in clinical translation during PCD‐mediated antitumor immune response.

Second, immunosuppressive TME will greatly affect antitumor efficacy. For example, T lymphocyte activation via PCD cascaded antitumor immunity can be alleviated by Tregs, MDSCs or TAMs. CAFs existence in TME supports tumorigenesis simultaneously impeding T cell infiltration, thereby weakening therapeutic efficacy. In addition, the high expression of PD‐1 on T cell surface contributes to tumor escape from host immunosurveillance via immune checkpoint. Thus, it will be better if nanotherapy strategies through eliciting tumor PCD for antitumor immune response combine with TME modulation and immune checkpoint blockade to achieve an enhanced antitumor efficacy.

Third, single approach may lead to drug resistance and insufficient antitumor efficacy. The rational combination therapy among different tumor PCD routes is necessary for an ideal synergistic efficacy. It does not mean a simple addition among approaches, while each component possesses a unique property for an amplified efficacy with a synergistic effect. The deeper relationships of PCD routes mediated antitumor immunity mechanisms should be explored for the combination therapy.

## Conflict of Interest

The authors declare no conflict of interest.
